# The Scoring Model to Predict ICU Stay and Mortality After Emergency Admissions in Atrial Fibrillation: A Retrospective Study of 30 366 Patients

**DOI:** 10.1002/clc.70101

**Published:** 2025-02-20

**Authors:** Tao Hong, Jian Huang, Jiewen Deng, Lirong Kuang, Mengyan Sun, Qianqian Wang, Chao Luo, Jikai Zhao, Xiaozhu Liu, Huishan Wang

**Affiliations:** ^1^ Postgraduate College Dalian Medical University Dalian China; ^2^ Department of Cardiovascular Surgery General Hospital of Northern Theater Command Shenyang China; ^3^ Department of Diagnostic Ultrasound Sir Run Run Shaw Hospital, Zhejiang University College of Medicine Hangzhou China; ^4^ Department of Neurosurgery Xiushan People's Hospital Chongqing China; ^5^ Department of Ophthalmology Wuhan Wuchang Hospital (Wuchang Hospital Affiliated to Wuhan University of Science and Technology) Wuhan China; ^6^ Harris Manchester College Oxford UK; ^7^ College of Medical Informatics Chongqing Medical University Chongqing China; ^8^ The People's Hospital of Shayang County Jingmen China; ^9^ Emergency and Critical Care Medical Center, Beijing Shijitan Hospital Capital Medical University Beijing China

**Keywords:** atrial fibrillation, emergency triage, machine learning, scoring model

## Abstract

**Background:**

The rapid assessment of the conditions is crucial for the prognosis of atrial fibrillation (AF) patients admitted to the emergency department (ED). We aim to derive and validate a more accurate and simplified scoring model to optimize the triage of AF patients in the ED.

**Materials and Methods:**

We conducted a retrospective study using data from the Medical Information Mart for Intensive Care (MIMIC‐IV) database and developed scoring models employing the Random Forest algorithm. The area under the receiver operating characteristic (ROC) curve (AUC) was used to measure the performance of the prediction for intensive care unit (ICU) stay, and the death likelihood within 3, 7, and 30 days following the ED admission.

**Results:**

The study included 30 366 AF patients, randomly divided into training, validation, and testing cohorts at a 7:1:2 ratio. The training set consisted of 21 257 patients, the validation set included 3036 patients, and the remaining 6073 patients were classified as the validation set. Among the cohorts, 9594 patients (32%) required ICU transfers, with mortality rates of 1% at 3 days, 3% at 7 days, and 6% at 30 days. In the testing set, the scoring models demonstrated strong discriminative ability with AUCs of 0.724 for ICU stay, 0.782 for 3‐day mortality, 0.755 for 7‐day mortality, and 0.767 for 30‐day mortality.

**Conclusion:**

We derived and validated novel simplified scoring models with good discriminative performance to predict the likelihood of ICU stay, 3‐day, 7‐day, and 30‐day death in AF patients after ED admission.

## Graphical abstract

Schematic overview of the study. This graphic illustrates the development and validation of a simplified scoring model designed to predict outcomes for AF patients upon ED admission. The model was derived using data from 30 366 patients in the MIMIC‐IV database (2008–2019), with 54% of patients being female and an average age of 75 years. Key predictors included demographic characteristics, comorbidities, urgency factors, vital signs, and social factors. The data set was divided into training (21 257 patients), validation (3036 patients), and testing (6073 patients) cohorts. Outcome measures for the model included ICU transfers and mortality rates at 3, 7, and 30 days post‐admission. The model's performance was evaluated using the AutoScore framework, which showed strong discriminative ability.

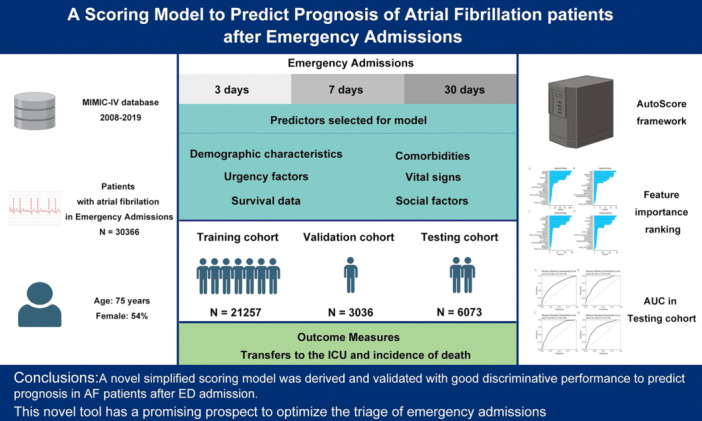

## 1 Introduction

Atrial fibrillation (AF) is the most common form of dysrhythmia encountered in patients presenting to the emergency department (ED) [1]. As the global population ages, the incidence of AF has risen correspondingly, leading to an increasing number of AF patients seeking care at EDs, which place additional strain on already limited emergency healthcare resources [2].

In overcrowded EDs, where patient volume is high, triage serves as the critical initial step for assessing and identifying high‐risk patients who require immediate attention [3]. This process is essential for the efficient allocation of finite ED resources and is a cornerstone of emergency medical systems. The quality of subsequent care, which is heavily influenced by the initial assessment of the patient's condition, plays a crucial role in determining prognosis [4, 5]. Therefore, triage systems capable of accurately predicting clinical outcomes are vital in guiding clinical decision‐making. Several scoring systems have been proposed to predict the prognosis of AF patients following ED admission [6, 7, 8], such as the AFTER study [6], which estimates the possibility of death within 30 days; the built TrOPs‐BAC model incorporated a positive troponin result, other acute ED diagnoses, pulmonary diseases, bleeding risk, 75 years of age or older, and congestive heart failure. However, previous studies often overlooked the inclusion of vital signs recorded at the time of ED admission (e.g., heart rate, respiratory rate, and mode of arrival), leaving the prediction of short‐term outcomes incompletely explored [9, 10, 11].

Machine learning algorithms, as a cutting‐edge tool in artificial intelligence, possess the ability to capture complex, nonlinear interactions more effectively than traditional statistical methods [12, 13]. This capability has demonstrated superior predictive performance in areas such as disease diagnosis and prognosis [14, 15]. Despite the potential benefits, the application of machine learning models to predict clinical outcomes for AF patients in the ED has been scarcely studied.

To address these gaps, we leveraged the Medical Information Mart for Intensive Care IV (MIMIC‐IV) database to develop machine learning‐based scoring models that accurately predict clinical outcomes for AF patients following ED admission. This approach could significantly enhance patient risk stratification and optimize triage processes.

## 2 Materials and Methods

### 2.1 Data Source

The data presented in this retrospective study was obtained from MIMIC‐IV. The database contains medical records from patients who were admitted to Beth Israel Deaconess Medical Center's Intensive Care Unit (ICU) or ED during the period from 2008 to 2019, and these records have undergone a process of reorganization and de‐identification [16]. The utilization of this database has greatly contributed to the development and application of machine learning algorithms in medical data analysis [17]. The research resource was reviewed and approved by the Institutional Review Board at the Beth Israel Deaconess Medical Center and the Massachusetts Institute of Technology. The board granted an exemption from the requirement for informed consent and approved the data‐sharing initiative.

### 2.2 Study Population and Variable Extraction

The study population consisted of patients identified in the MIMIC‐IV database between 2008 and 2019. The inclusion criteria were as follows: adult patients (aged ≥ 18 years) with AF at the time of ED, defined as the International Classification of Diseases, 9th Version (ICD‐9) codes of 427.31 or ICD‐10 codes of I480, I481, I482, and I4891. Subsequently, all eligible patients were randomly distributed, with 70% assigned to the training set, 10% to the validation set, and the remaining 20% to the testing set. We extracted the following baseline characteristics from the data: demographic characteristics, vital signs, comorbidities, severity and urgency, socioeconomic status, and survival data. The demographic information of the patients included their age and gender. Vital signs, which are important indicators of patient health, consist of temperature, heart rate, respiratory rate, oxygen saturation (O2sat), systolic blood pressure (SBP), and diastolic blood pressure (DBP). The comorbidities that were considered and could potentially impact outcomes included hypertension, ischemic heart disease, cerebrovascular disease, dementia, chronic pulmonary disease, diabetes, renal disease, severe liver disease, and cancer. Patients with these comorbidities are often severely ill, and some even die within 1 day. The urgency of the patients was assessed by considering factors including the mode of arrival (walking, ambulance, helicopter), pain score, and intubation status. Survival data included ICU stay, 3‐day mortality, 7‐day mortality, and 30‐day mortality. Additionally, we evaluated the social factors of the patients including their insurance status, as previous studies have suggested that socioeconomic status can affect the post‐emergency prognosis of patients [18].

### 2.3 Outcome Measures

The primary endpoints for the development and evaluation of the scoring models were transfers to the ICU and incidence of death within 3 days. The criterion for 3‐day death was fatalities that occurred within 72 h after admission. Secondary endpoints include mortality within 7 days and 30 days after ED admission, including deaths during hospitalization and after discharge from ED and hospital. All this information was documented within the MIMIC‐IV database.

### 2.4 Statistical Analysis

In the training set (70% randomly selected samples), variables were initially ranked based on their feature importance to identify variables (referred to as features in machine learning terminology) from largest to smallest contribution to the machine's prediction [19]. Given the Random Forest (RF) algorithm's significant advantages over other methodologies in handling highly nonlinear correlated data, its robustness against noise, simplicity in tuning, potential for efficient parallel processing, and its ability to reduce the variable space by giving an importance value to each feature [20], we chose the RF algorithm to select the variables for the model based on their impact on the area under the receiver operating characteristic curve (AUC) values. The validation set (the randomly 10% sample) was used to assess parameter tuning and identify the best‐performing candidate scoring models. Four scoring models were ultimately derived to predict the likelihood of ICU stay, as well as death within 3 days, 7 days, and 30 days. Finally, the prediction power of each derived model was evaluated in the testing set (the remaining 20% sample), with an AUC value exceeding 0.7 considered indicative of an acceptable predictive model. We calculated sensitivity, specificity, positive predictive value (PPV), and negative predictive value (NPV) under the optimal threshold and reported them with 95% confidence intervals (95% CI). All statistical analyses were performed using R software (version 4.2.3). In the depiction of patients’ baseline characteristics, continuous parameters were expressed as the median with quartile (Q1, Q3) for non‐normal distributions, while categorical data were presented as numbers with percentages. Values of vital signs that were beyond the range of clinical and physiological cognition were considered outliers. In the analysis, we used K‐Nearest Neighbors to interpolate the missing values.

AutoScore is an automatic clinical score generator based on machine learning that can be used to derive scoring models [21]. The initial scoring model was developed using the AutoScore framework using the training set. Parameter tuning and model selection for potential scoring models were evaluated using the validation set. Ultimately, four scoring models were developed to forecast the likelihood of ICU stay, as well as death within 3 days, 7 days, and 30 days, respectively. The testing cohort was used to analyze the performance metrics of the final scoring models. Furthermore, the four scoring models were employed to predict the chance of ICU transfer following emergency admission in the testing set, and their performance was assessed. Statistical significance was considered with a p‐value below 0.05.

## 3 Results

### 3.1 Patient Characteristics

**FIGURE 1:**
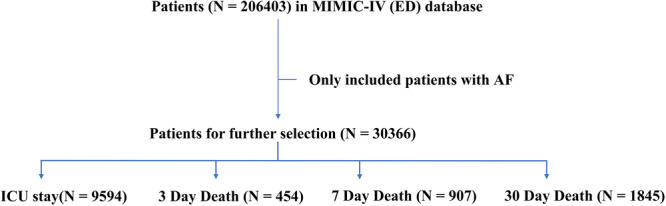
Patient screening flowchart.

A total of 206 403 patients who visited the emergency department were identified from the MIMIC‐IV database, we included a final sample of 30 366 patients diagnosed with AF, as indicated in the flowchart in Figure 1. The median age of the patients was 75 years, with 16 259 (54%) of them being female. Then all subjects were allocated to the training, validation, and testing sets in a ratio of 7:1:2. Of these, 21 257 patients with a median age of 75 years were randomly assigned to the training set of whom 11 393 (54%) were female. In addition, the validation set included 3036 patients with a mean age of 75 years, of whom 1618 (53%) were female. Ultimately, 6073 patients with an average age of 75 years were assigned to the testing set, of whom 3248 (53%) were female. Overall, of the 30 366 medical records reviewed, 9594 (32%) patients were admitted to the ICU after presenting to the ED. Observed mortality for the entire cohort was 1% (454 cases) at 3 days, 3% (907 cases) at 7 days, and 6% (1845 cases) at 30 days. As depicted in Table 1, there were no statistically significant differences in comparing baseline characteristics of training, validation, and testing tests, including variables such as age, sex, vital signs, and comorbidities.

**TABLE 1:** Characteristics of the study patients.

Variables	Total (*n* = 30 366)	Training set (*n* = 21 257)	Validation set (*n* = 3036)	Testing set (*n* = 6073)	*p*
Age, Median (Q1, Q3)	75 (65, 83)	75 (65, 83)	75 (65, 83)	75 (66, 83)	0.732
Gender, n (%)					0.947
Female	16 259 (54)	11 393 (54)	1618 (53)	3248 (53)	
Male	14 107 (46)	9864 (46)	1418 (47)	2825 (47)	
Hypertension, n (%)					0.659
No	6454 (21)	4537 (21)	626 (21)	1291 (21)	
Yes	23912 (79)	16 720 (79)	2410 (79)	4782 (79)	
IHD, n (%)					0.911
No	26 209 (86)	18 338 (86)	2619 (86)	5252 (86)	
Yes	4157 (14)	2919 (14)	417 (14)	821 (14)	
Heart. failure, n (%)					0.28
No	20 797 (68)	14 613 (69)	2074 (68)	4110 (68)	
Yes	9569 (32)	6644 (31)	962 (32)	1963 (32)	
Cerebrovascular_disease, n (%)					0.658
No	26 089 (86)	18 258 (86)	2596 (86)	5235 (86)	
Yes	4277 (14)	2999 (14)	440 (14)	838 (14)	
Dementia, n (%)					0.583
No	27 952 (92)	19 545 (92)	2800 (92)	5607 (92)	
Yes	2414 (8)	1712 (8)	236 (8)	466 (8)	
Chronic_pulmonary_disease, n (%)					0.613
No	21 881 (72)	15 300 (72)	2176 (72)	4405 (73)	
Yes	8485 (28)	5957 (28)	860 (28)	1668 (27)	
Diabetes, n (%)					0.258
No	19 864 (65)	13 884 (65)	1960 (65)	4020 (66)	
Yes	10 502 (35)	7373 (35)	1076 (35)	2053 (34)	
Renal_disease, n (%)					0.769
No	19 958 (66)	13 997 (66)	1992 (66)	3969 (65)	
Yes	10 408 (34)	7260 (34)	1044 (34)	2104 (35)	
Severe_liver_disease, n (%)					0.64
No	29 751 (98)	20 816 (98)	2977 (98)	5958 (98)	
Yes	615 (2)	441 (2)	59 (2)	115 (2)	
Cancer, n (%)					0.742
No	26 889 (89)	18 810 (88)	2701 (89)	5378 (89)	
Yes	3477 (11)	2447 (12)	335 (11)	695 (11)	
Insurance, n (%)					0.853
Medicare	19 228 (63)	13 438 (63)	1921 (63)	3869 (64)	
Medicaid	733 (2)	525 (2)	72 (2)	136 (2)	
Others	10 405 (34)	7294 (34)	1043 (34)	2068 (34)	
Arrival_transport, n (%)					0.753
Walk in	9835 (32)	6882 (32)	972 (32)	1981 (33)	
Ambulance	20 297 (67)	14 205 (67)	2046 (67)	4046 (67)	
Helicopter	234 (1)	170 (1)	18 (1)	46 (1)	
Temperature, Median (Q1,Q3)	36.8 (36.7, 37.1)	36.8 (36.7, 37.1)	36.8 (36.7, 37.1)	36.9 (36.7, 37.1)	0.449
Heartrate, Median (Q1,Q3)	89 (77, 106)	89 (76, 106)	89 (77, 106)	89 (77, 106)	0.906
Respirate, Median (Q1,Q3)	20 (18, 24)	20 (18, 24)	20 (18, 24)	20 (18, 24)	0.647
O2sat, Median (Q1,Q3)	99 (98, 100)	99 (98, 100)	99 (98, 100)	99 (98, 100)	0.19
Sbp, Median (Q1,Q3)	141 (126, 157)	141 (125, 157)	141 (126, 157)	142 (126, 157)	0.414
Dbp, Median (Q1,Q3)	81 (71, 92)	81 (71, 92)	81 (71, 92)	81 (71, 92)	0.257
Pain, Median (Q1,Q3)	0 (0, 5)	0 (0, 5)	0 (0, 5)	0 (0, 5)	0.905
Intuabted, n (%)					0.386
No	30 299 (100)	21 207 (100)	3028 (100)	6064 (100)	
Yes	67 (0)	50 (0)	8 (0)	9 (0)	
ICU_stay, n (%)					0.145
No	20 772 (68)	14 470 (68)	2091 (69)	4211 (69)	
Yes	9594 (32)	6787 (32)	945 (31)	1862 (31)	
3‐day death, n (%)					0.71
No	29 912 (99)	20 932 (98)	2991 (99)	5989 (99)	
Yes	454 (1)	325 (2)	45 (1)	84 (1)	
7‐day death, n (%)					0.694
No	29 459 (97)	20 611 (97)	2951 (97)	5897 (97)	
Yes	907 (3)	646 (3)	85 (3)	176 (3)	
30‐day death, n (%)					0.186
No	28 521 (94)	19 933 (94)	2855 (94)	5733 (94)	
Yes	1845 (6)	1324 (6)	181 (6)	340 (6)	

Abbreviations: DBP, diastolic blood pressure; ICU, intensive care unit; IHD, ischemic heart disease; SBP, systolic blood pressure.

### 3.2 Variable Selection and Scoring Model Establishment

We used feature importance to rank the relative importance of all collected 22 features. This clarifies which predictors are the most important for determining prognosis in AF. We found that the following variables consistently remained in the top five rankings for all four outcomes: SBP, heart rate, temperature, and DBP. It's worth noting that the specific rankings of these variables may vary for each outcome (Figure S1A–D).

Afterward, we employed the Random Forest algorithm to assess the relationship between the selected variables and the corresponding changes in AUC values. The goal of this was to determine which predictors were required to achieve an acceptable accuracy to deploy a clinically feasible scoring model. Subsequently, we narrowed down the number of variables to 11 to construct our model and ensure the predictive performance of the model. The 11 variables (respirate, heart rate, SBP, DBP, temperature, age, pain, O2sat, arrival transport, insurance, and cerebrovascular disease) were used for predicting ICU stay, 7‐day death, and 30‐day death. cerebrovascular disease hasn't been included in the 3‐day death mode. Building upon this foundation, other variables can't improve the performance of the scoring models (Figure S2A–D).

The ICU‐stay scoring model is shown in Table 2. The scores are predominantly influenced by the following factors: respiratory rate ≥ 31 beats per minute (bpm), heart rate ≥ 111 bpm, SBP < 90 mmHg, temperature < 36.4°C, DBP < 60 mmHg, age < 80 years old, pain score ≥ 6, O2sat < 96, other insurance, the arrival transport of helicopter, and cerebrovascular disease. In the 3‐day death scoring model, the scores are primarily determined by respiratory rate ≥ 24 beats per minute (bpm), heart rate ≥ 110 bpm, SBP < 90 mmHg, temperature < 36.5°C, DBP < 60 mmHg, age ≥ 85 years old, pain score < 6, O2sat < 97, others insurance, and the arrival transport of helicopter. In the 7‐day death scoring model, the scores are primarily contributed by respiratory rate ≥ 24 beats per minute (bpm), heart rate ≥ 110 bpm, SBP < 90 mmHg, temperature < 36.3°C, DBP < 60 mmHg, age ≥ 83 years old, pain score < 5, O2sat < 98, Medicaid insurance, the arrival transport of helicopter and cerebrovascular disease. In the 30‐day death scoring model, the scores are primarily contributed by respiratory rate ≥ 24 beats per minute (bpm), heart rate ≥ 111 bpm, SBP < 90 mmHg, temperature < 36°C, DBP < 50 mmHg, age ≥ 85 years old, the arrival transport of helicopter and cerebrovascular disease.

**TABLE 2:** Scoring models for the ICU stay, 3‐day death, 7‐day death, and 30‐day death.

ICU stay			3‐day		7‐day		30‐day	
variable	interval	point	interval	point	interval	point	interval	point
Respiration	< 16	5	< 20	0	< 20	0	< 18	3
	[16,18)	0	[20, 24)	1	[20,24)	3	[18,20)	0
	[18,24)	2	≥ 24	7	≥ 24	9	[20,24)	5
	[24,31)	10					≥ 24	10
	≥ 31	18						
Heart rate	< 62	0	< 60	2	< 60	2	< 90	0
	[62,74)	0	[60, 90)	0	[60,90)	0	[90,100)	4
	[74,111)	3	[90, 110)	6	[90,110)	5	[100,111)	6
	[111,142)	7	≥ 110	8	≥ 110	7	≥ 111	7
	≥ 142	8						
SBP	< 90	26	< 90	26	< 90	25	< 90	23
	≥ 90	0	[90, 120)	8	[90,135)	5	[90,110)	10
			[120, 140)	4	≥ 135	0	[110,120)	5
			[140, 160)	2			[120,140)	5
			≥ 160	0			≥ 140	0
Temperature	< 36.4	8	< 36.5	10	< 36.3	13	< 36	15
	[36.4,36.6)	3	[36.5, 37.3)	2	[36.3,37)	0	[36,36.3)	9
	[36.6,37.2)	0	≥ 37.3	0	≥ 37	0	[36.3,37)	0
	[37.2,38.3)	2					[37,37.3)	1
	≥ 38.3	5					≥ 37.3	1
DBP	< 60	6	< 60	6	< 60	4	< 50	10
	[60,90)	2	[60,80)	1	[60,90)	1	[50,60)	3
	> = 90	0	≥ 80	0	≥ 90	0	[60,70)	2
							≥ 70	0
Age	< 80	2	< 56	0	< 63	0	< 63	0
	≥ 80	0	[56, 63)	1	[63,75)	4	[63,71)	3
		2	[63, 71)	3	[75,83)	7	[71,85)	6
		0	[71, 85)	7	≥ 83	11	≥ 85	10
			≥ 85	11				
Pain score	< 6	0	< 6	1	< 5	2	< 6	0
	≥ 6	1	≥ 6	0	≥ 5	0	≥ 6	0
O2sat	< 96	3	< 97	6	< 98	1		
	[96,98)	0	[97,98)	1	[98,99)	0	< 98	0
	≥ 98	2	[98,99)	3	≥ 99	0	[98,99)	0
			≥ 99	0			≥ 99	0
Arrival transport	Walk in	0	Walk in	0	Walk in	0	Walk in	0
	Ambulance	7	Ambulance	8	Ambulance	6	Ambulance	6
	Helicopter	21	Helicopter	22	Helicopter	16	Helicopter	15
Insurance	Medicare	0	Medicare	0	Medicare	0	Medicare	0
	Medicaid	0	Medicaid	1	Medicaid	4	Medicaid	1
	Others	1	Others	4	Others	1	Others	1
Cerebrovascular disease	No	0			No	0	No	0
	Yes	8			Yes	8	Yes	8

Abbreviations: DBP, diastolic blood pressure; SBP, systolic blood pressure.

Combined with RF algorithms, AutoScore automatically develops a minimalistic sparse score risk model for predefined outcomes, enabling users to quickly and seamlessly build interpretable clinical scores that can be easily implemented and validated in clinical practice. Table S1 displays the various predicted risk probabilities, while Table 3 presents the different predicted scores.

**TABLE 3:** Varying score cut‐offs of predicted risk based on the scoring models of ICU stay, 3‐day death, 7‐day death, and 30‐day death in the testing cohort.

Outcome	Score cut‐off [≥]	Predicted Risk [≥]	Percentage of patients (%)	Accuracy (95% CI)	Sensitivity (95% CI)	Specificity (95% CI)	PPV (95% CI)	NPV (95% CI)
ICU stay	5	7.90%	99	31.2% (31%–31.4%)	99.8% (99.6%–100%)	0.8% (0.5%–1.1%)	30.8% (30.7%–30.9%)	92.1% (81.8%–100%)
	15	19.80%	72	50.2% (49.2%–51.3%)	86.8% (85.2%–88.3%)	34.1% (32.7%–35.5%)	36.8% (36.1%–37.4%)	85.4% (83.8%–86.9%)
	25	41.50%	25	72.8% (71.8%–73.7%)	46.4% (44.1%–48.6%)	84.4% (83.4%–85.5%)	56.9% (54.9%–58.9%)	78.1% (77.3%–78.8%)
	35	67.10%	6	72.8% (72.2%–73.3%)	14.9% (13.3%–16.5%)	98.3% (98%–98.7%)	79.9% (75.7%–83.9%)	72.3% (71.9%–72.7%)
	45	85.50%	1	70% (69.8%–70.3%)	2.5% (1.8%–3.3%)	99.9% (99.7%–100%)	89.1% (78.9%–96.6%)	69.8% (69.7%–70%)
	50	90.90%	0	69.6% (69.4%–69.7%)	0.8% (0.4%–1.2%)	100% (99.9%–100%)	88.9% (70.6%–100%)	69.5% (69.4%–69.6%)
3‐day death	5	0.10%	100	1.6% (1.5%–1.7%)	100% (100%–100%)	0.2% (0.1%–0.4%)	1.4% (1.4%–1.4%)	100% (100%–100%)
	15	0.20%	91	10.1% (9.4%–10.8%)	100% (100%–100%)	8.8% (8.2%–9.6%)	1.5% (1.5%–1.5%)	100% (100%–100%)
	25	0.60%	59	42.3% (41%–43.4%)	90.5% (84.5%–96.4%)	41.6% (40.3%–42.8%)	2.1% (2%–2.3%)	99.7% (99.5%–99.9%)
	35	2%	20	79.9% (78.9%–80.9%)	58.3% (47.6%–69%)	80.2% (79.1%–81.2%)	4% (3.2%–4.6%)	99.3% (99.1%–99.5%)
	45	5.90%	3	96.4% (96%–96.8%)	22.6% (14.3%–31%)	97.4% (97%–97.8%)	11% (7%–15.3%)	98.9% (98.8%–99%)
	55	16.30%	0	98.6% (98.4%–98.7%)	10.7% (4.8%–17.9%)	99.8% (99.7%–99.9%)	40.7% (20.7%–62.5%)	98.8% (98.7%–98.9%)
7‐day death	15	0.70%	82	21% (20%–22%)	99.4% (98.3%–100%)	18.7% (17.6%–19.7%)	3.5% (3.5%–3.6%)	99.9% (99.7%–100%)
	20	1.20%	63	39.4% (38.2%–40.6%)	94.9% (91.5%–97.7%)	37.7% (36.5%–39%)	4.4% (4.2%–4.5%)	99.6% (99.3%–99.8%)
	25	2.10%	41	60.6% (59.4%–61.8%)	86.4% (81.2%–91.5%)	59.9% (58.6%–61.1%)	6% (5.7%–6.4%)	99.3% (99.1%–99.6%)
	35	6.70%	11	88.8% (88%–89.6%)	39.8% (32.9%–47.2%)	90.3% (89.5%–91%)	10.9% (9%–12.9%)	98% (97.8%–98.3%)
	40	11.50%	4	94.5% (94.1%–95%)	18.8% (13.1%–24.4%)	96.8% (96.4%–97.3%)	14.9% (10.8%–19.2%)	97.6% (97.4%–97.7%)
	45	19%	1	96.5% (96.2%–96.8%)	9.7% (5.7%–14.2%)	99.1% (98.9%–99.3%)	24.1% (15.2%–34%)	97.3% (97.2%–97.5%)
30‐day death	5	0.60%	99	6.7% (6.5%–7%)	100% (100%–100%)	1.2% (0.9%–1.5%)	5.7% (5.6%–5.7%)	100% (100%–100%)
	15	1.80%	80	24.9% (23.8%–25.9%)	95.9% (93.8%–97.6%)	20.7% (19.6%–21.7%)	6.7% (6.5%–6.8%)	98.8% (98.2%–99.4%)
	25	4.90%	40	63.6% (62.3%–64.7%)	78.2% (74.1%–82.4%)	62.7% (61.4%–63.9%)	11.1% (10.5%–11.7%)	98% (97.6%–98.4%)
	35	12.90%	9	88.5% (87.7%–89.2%)	30.3% (25.6%–35.6%)	91.9% (91.2%–92.6%)	18.2% (15.4%–21.1%)	95.7% (95.4%–96%)
	45	29.60%	1	94% (93.7%–94.2%)	5.9% (3.5%–8.5%)	99.2% (98.9%–99.4%)	29.9% (19.3%–41%)	94.7% (94.5%–94.8%)
	55	54.40%	0	94.5% (94.3%–94.6%)	2.4% (0.9%–4.1%)	99.9% (99.8%–100%)	61.9% (33.3%–88.2%)	94.5% (94.4%–94.6%)

Abbreviations: NPV, negative predictive values; PPV, positive predictive values.

These tables demonstrated the proportion of patients and performance estimates observed in the test set for each scoring model. According to our scoring model, the predicted risk probability for ICU admission ranges from 7.9% to 90.9%, which is higher than the other three groups (0.1%–54.4%). This difference arises because 32% of patients in the entire cohort require further supportive treatment in the ICU, whereas the proportion of patients assigned to the other three groups is lower. Moreover, the prediction model for each outcome had corresponding cutoff values, the best score threshold of ICU stay, 3‐day death, 7‐day death, and 30‐day death are 20, 33, 30, and 25, respectively. With the increase of the model score, the probability of the outcome event also increases. Moreover, the probability increases by a multiple that is higher than the score increase, for example, in the ICU stay group, when the score is five points, the probability is 7.9%, when the score is increased by three times, the probability increases by 2.5 times, but when the score is increased by seven times, the probability increases by 8.5 times, which shows that the high score does represent the critical situation of the disease. This phenomenon was more pronounced in the 3‐day, 7‐day, and 30‐day death groups. For example, in the 3‐day death group, the score increased from five points to 35 points, but the probability increased by 20‐fold. Because patients with low scores had a low probability of dying, but with the increase in scores, the likelihood of malignant events increased greatly.

### 3.3 Performance Evaluation

**FIGURE 2:**
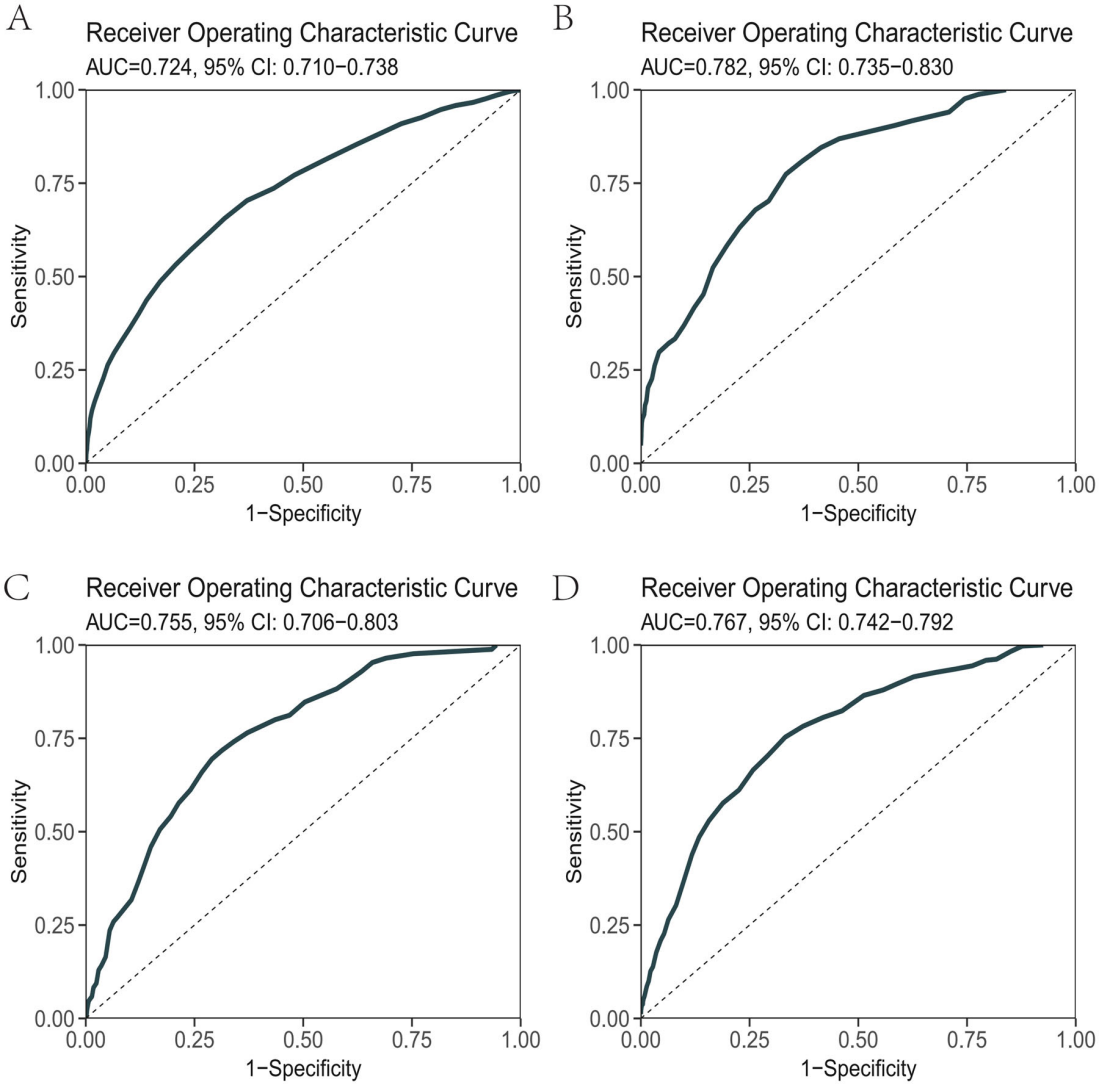
AUC values of the ICU stay (A), 3‐day death (B), 7‐day death (C), and 30‐day death (D) in the testing set.

We evaluated the predictive performance of the scoring models for ICU admission, 3‐day mortality, 7‐day mortality, and 30‐day mortality. The estimation results for each cohort are summarized in Figures 2A–D, S3A–D, and S4A–D. In both the training set and the validation set, most of the AUC values were higher than 0.75, indicating that our model has high accuracy, which was also verified by the testing set. Across the testing cohorts, the scoring model performed well in predicting short‐ and long‐term mortality, with AUCs of 0.724 (95% CI 0.710–0.738) for ICU stay, 0.782 (95% CI 0.735–0.830) for death at 3 days, 0.755 (95% CI 0.706–0.803) for death at 7 days, and 0.767 (95% CI 0.742–0.792) for death at 30 days. Among four outcomes, the scoring model demonstrated its highest predictive performance in mortality within 3 days. Meanwhile, the calibration curves for the training cohort and the testing cohort revealed the predicted probability is in good agreement with the actual occurrence. (Figures S5A–D and S6). The calibration curves reveal that for the ICU stay model, the calibration curve is closely aligned with the diagonal, with minimal fluctuation in the observed values, indicating that the predicted probabilities are in strong agreement with the actual outcomes (Figure S6A). In contrast, the calibration curves for the 3‐day, 7‐day, and 30‐day prediction models show slight deviations (Figure S6B–D). This may be due to the higher accuracy of ED variables in predicting short‐term outcomes. However, as time progresses and treatment interventions affect patient conditions, discrepancies arise between predicted probabilities and actual outcomes.

## 4 Discussion

In this retrospective study involving 30 366 AF patients who presented to the ED, we developed novel risk‐scoring models using RF algorithm to identify patients with an urgent need for ICU admission and those at risk of mortality within 3, 7, and 30 days. To the best of our knowledge, this is the largest study to date focused on predicting short‐term outcomes in AF patients presenting to the ED. The risk‐scoring models demonstrated strong discriminatory performance, selecting 10–11 key features for score calculation. These variables include respirate, heart rate, SBP, DBP, temperature, age, pain, O2sat, arrival transport, insurance, and cerebrovascular disease. The concise and precise risk‐scoring models developed in this study can effectively identify high‐risk AF patients in the ED, thereby improving patient safety and optimizing ED triage processes.

AF continues to pose a significant public health challenge and economic burden. Its prevalence is steadily increasing and is projected to reach 15.9 million cases by 2050 [22]. In addition to increasing the risk of thromboembolism and congestive heart failure, AF is also associated with higher overall and cardiovascular mortality [8, 23]. In our study, we observed that 32% of AF patients were transferred to the ICU after presenting to the ED, with mortality rates of 1%, 3%, and 6% within 3, 7, and 30 days, respectively. Thus, the impact of conditions resulting from AF is indeed significant.

Meanwhile, the rising incidence of AF further strains ED resources. In the fast‐paced and ever‐changing ED environment, one of the most challenging tasks is to determine whether a patient may deteriorate and how quickly this may happen [24]. Consequently, ED physicians must reliably identify high‐risk individuals, enabling the safe discharge of more patients and the efficient management of a larger number of cases in a shorter timeframe [16, 25].

Given this situation, previous studies have developed various risk stratification models to predict ICU admission and death in the ED [26, 27, 28, 29, 30]. However, there is a paucity of predictive models regarding the prognosis of AF in the ED. Atzema et al. conducted the “AFTER” and “AFTER2” studies using a cohort of 3510 emergency room patients with AF [6, 7]. These studies aimed to predict 30‐day mortality and cardiovascular hospitalization, respectively. However, both studies employed logistic regression models and were limited by a relatively small sample size. In this present study, we employed the largest study cohort of AF in ED to date and analyzed a broader range of short‐term outcomes. This approach enables a more objective and comprehensive assessment of the progression of AF patients’ conditions in the ED.

Furthermore, machine learning algorithms have been increasingly implemented in the field of emergency medicine [12, 13, 14, 15, 16, 30]. Raita et al. developed four machine learning models (Lasso Regression, Random Forest, Gradient Boosted Decision Tree, and Deep Neural Network) using 135 470 eligible ED visits, which demonstrated superior performance in predicting critical care and hospitalization outcomes compared with that of a conventional method—the Emergency Severity Index (ESI) [12]. Klug et al. applied a Gradient Boosting machine learning model for predicting mortality in the ED, achieving high predictive accuracy with an AUC of 0.962 for early mortality (within 2 days) and 0.923 for short‐term mortality (2–30 days) [14]. Lee et al. developed an artificial intelligence model (combined the three best models: Adaptive Boosting, Extreme Gradient Boosting, and Light Gradient Boosting) to predict trauma mortality that exhibited remarkable accuracy in predicting ED mortality with an AUROC of 0.9974 [30]. Machine learning algorithms have distinct advantages in processing large datasets of electronic medical records and extracting valuable patterns from vast amounts of data [31]. However, the application of machine learning algorithms to AF patients presenting to the ED has not been extensively explored. Although in Samaras and colleagues’ study, the RF algorithms were used to predict all‐cause mortality in hospitalized patients with concurrent AF, resulting in a high C‐index of 0.85 [8], but they focused on a 2‐year period, none has used machine learning algorithms to investigate the early and short‐term mortality of AF patients visited at the ED. This is precisely what we aim to explore in our study.

In addition, the previously reported scoring systems have complex requirements for variables [6, 7, 8, 18], such as laboratory and imaging results. And the MIMIC IV database lacks certain useful clinical variables (medications, socioeconomic status, international normalized ratio, troponin result, and creatinine level). However, the primary objective of this study is not to incorporate an extensive set of predictors but rather variables readily available in emergency situations to construct predictive models at current ED triage. In our study, both the potential variables and the variables eventually chosen for the scoring models can be easily collected in the initial phase upon ED arrival. This enhances the practicality of this clinical tool, highlighting the advantage of a simple scoring system.

It is worth noting that the final chosen variables and their corresponding scoring trends align well with clinical practice. In our scoring model, patients with rapid respiration, elevated heart rate, lower oxygen saturation, lower SBP and DBP, the way of transport, and lower body temperature were indicative of a higher likelihood of developing adverse outcomes and were therefore assigned higher scores. Shortness of respiration often represents the hypoxic state of the patient [32], which is a recognized emergency feature of the disease and was included in our four outcomes, and the assigned scores of different respiratory rates are also different. As indicated in Table 3, in the ICU stay model, when the respiratory rate reaches more than 31 breaths per minute, the score is 18 points. Only this variable alone, without considering other variables, obtained a 19.8% probability of ICU stay. Patients with low blood pressure are often frail and have insufficient tissue perfusion, which is more likely to be associated with a worse prognosis [33]. Xu et al. have revealed that having a baseline SBP ≤ 110 mmHg or DBP < 70 mmHg was associated with a significantly increased risk of all‐cause mortality in patients with AF [34]. In addition, we also included the way of transportation for ED visits, patients arriving in the ED by helicopter may be in extremely critical condition and must be treated immediately [35], so they are likely to be admitted to the ICU for further supportive care. In this context, if patients are inappropriately discharged or the standards for admission from the ED have become stricter, ED mortality rates may increase. Hence, ED costs deserve substantial attention. Interestingly, we observed that Medicare assigned zero score in all models, which indicate Medicare has no negative effects on the poor prognosis. Laura et al. previously reported that mortality within 30 days of Medicare beneficiaries receiving ED care have declined in recent years [36]. In addition, the thresholds were different for each variable for the four outcomes, reflecting the individualization of the model.

Our model quantifies the risk of mortality as a percentage, offering an objective measure that complements clinical assessments based on doctors’ judgment. This approach can enhance clinical decision‐making. For example, consider a 60‐year‐old patient with AF and cerebrovascular disease who arrives at the ED by helicopter. After a brief examination in the ED, the patient's heart rate is 95 bpm, respiratory rate is 28 bpm, SBP is 93 mmHg, DBP is 50 mmHg, O2sat is 95%, insurance is Medicaid, pain score is 5, and body temperature is 36.6 Celsius degrees. Based on the ICU stay scoring model, the patient is rated with a score of 53 and a probability of more than 90% to the ICU for further treatment. The likelihood of death within 3, 7, and 30 days is 16.3%, 19%, and 29.6%, respectively. Therefore, this patient warrants higher‐quality medical care

Our study has several limitations. Firstly, although we used a large population‐based sample for both derivation and validation, it is important to note that these were retrospective analyses, and the work capacity of the ICU admission was contingent on the local healthcare resource. The context in which the data were collected (including clinical practices, policies, and healthcare resources) has evolved over the extended period between 2008 and 2019, which presents a trade‐off for the large sample size (e.g., the transport pattern). Then, due to the limited data variables in the MIMIC‐IV database, we are unable to compare the predictive ability between our scoring models and previous predictive models. Moreover, during ED visits, vital signs are prone to rapid fluctuations. This can result in similar predictive outcomes for patients with different risk stratification, which potentially affecting the accuracy of the scoring system.

## 5 Conclusions

We have developed a novel clinical tool using machine learning algorithms to enhance triage for AF in the ED. The implementation of this advanced and concise scoring model demonstrates strong discriminatory ability in assessing the likelihood of AF patients being transferred to the ICU, as well as their risk of mortality at 3 days, 7 days, and 30 days after they visit the ED.

## Ethics Statement

The study received ethical oversight from the Institutional Ethics Committee of Chongqing Medical University.

## Conflicts of Interest

The authors declare no conflicts of interest.

## Supporting information

Supporting information.

Supporting information.

Supporting information.

Supporting information.

Supporting information.

Supporting information.

Supporting information.

Supporting information.

## Data Availability

The data will be made available upon reasonable request.
