# Supplementary figures and images for: The Scoring Model to Predict ICU Stay and Mortality After Emergency Admissions in Atrial Fibrillation: A Retrospective Study of 30 366 Patients
Source: Clin Cardiol. 2025 Feb 20;48(2):e70101. doi: 10.1002/clc.70101 (PMC11841604; doi:10.1002/clc.70101)

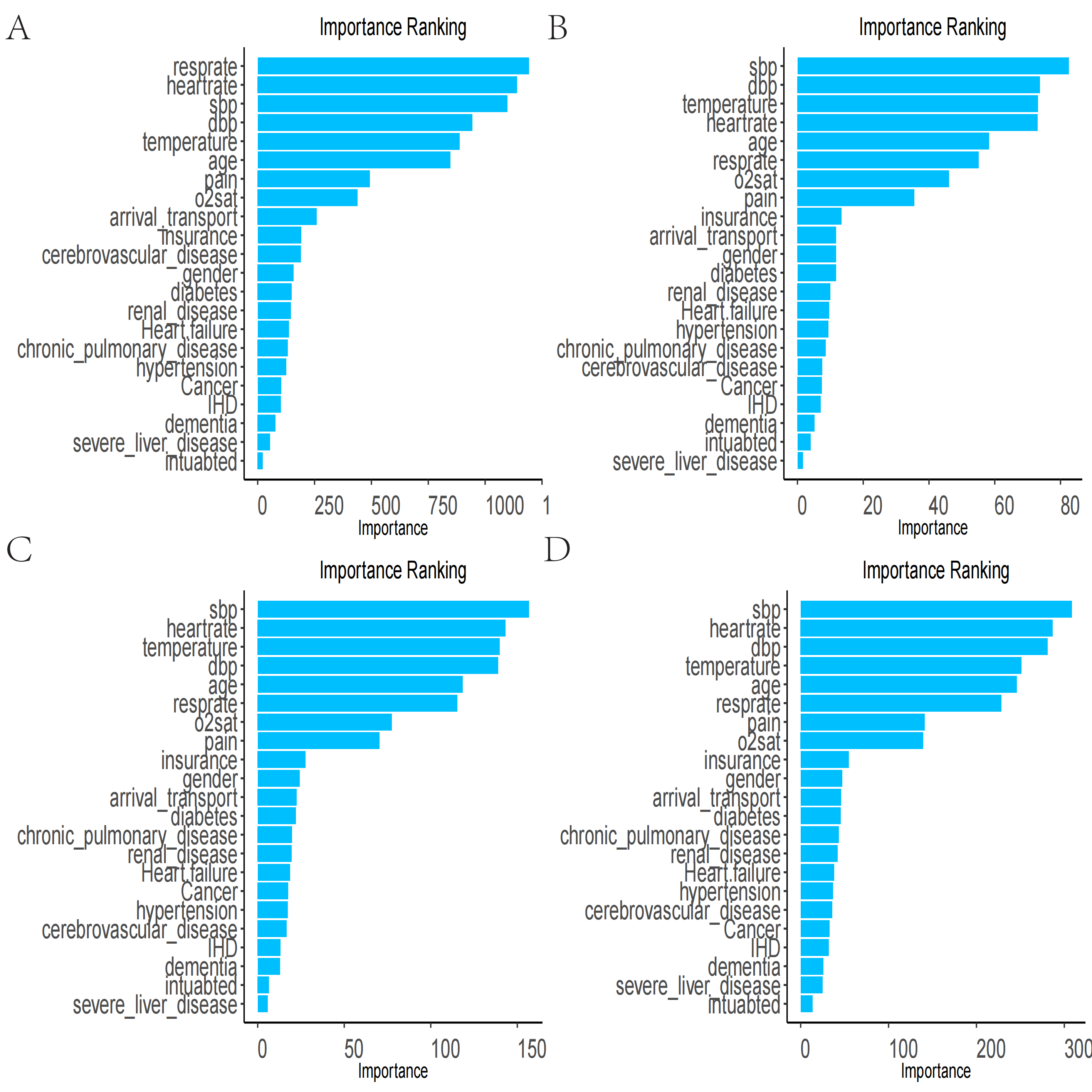

Supplement: Supplementary file 1 — Supporting information. [file CLC-48-e70101-s005.png]

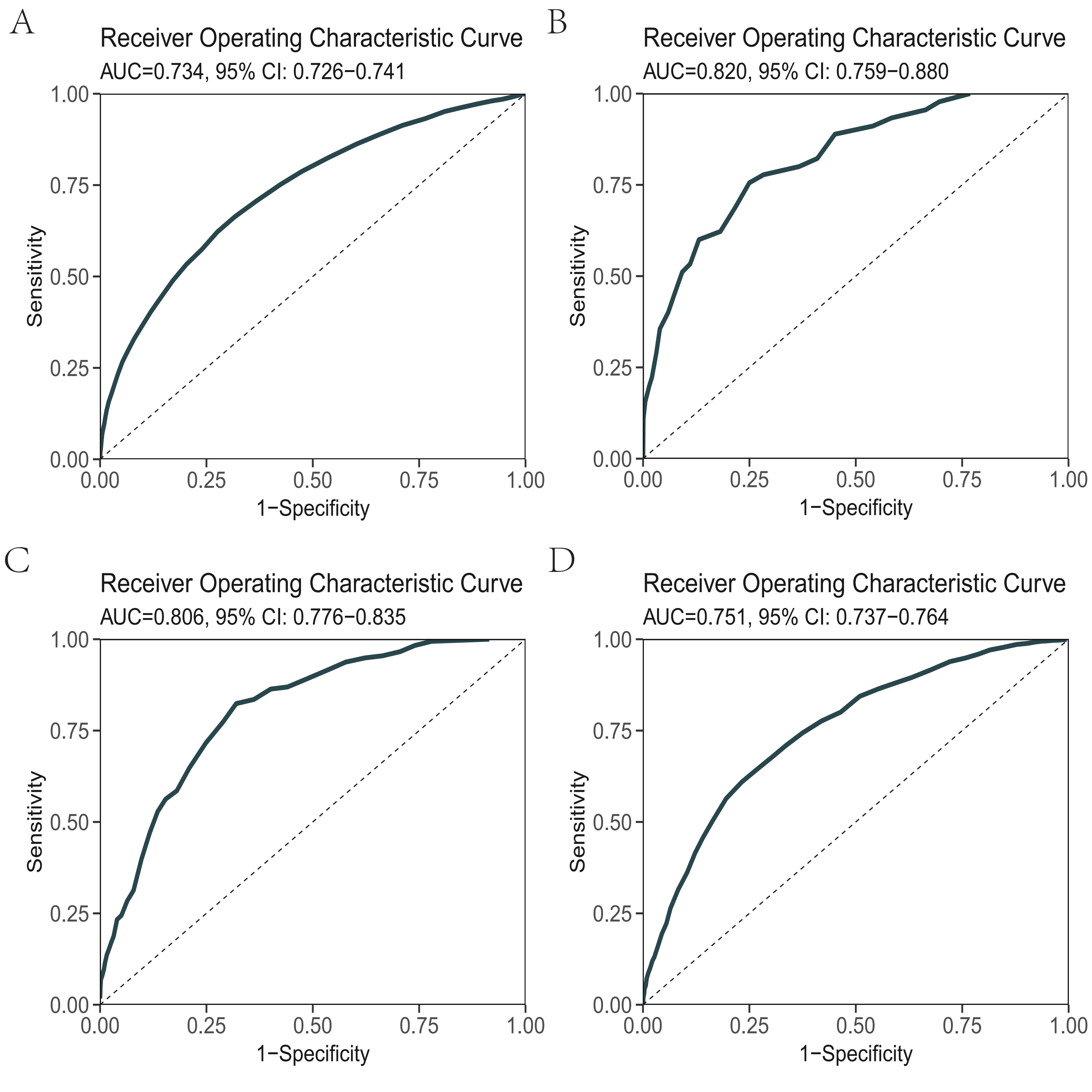

Supplement: Supplementary file 3 — Supporting information. [file CLC-48-e70101-s002.png]

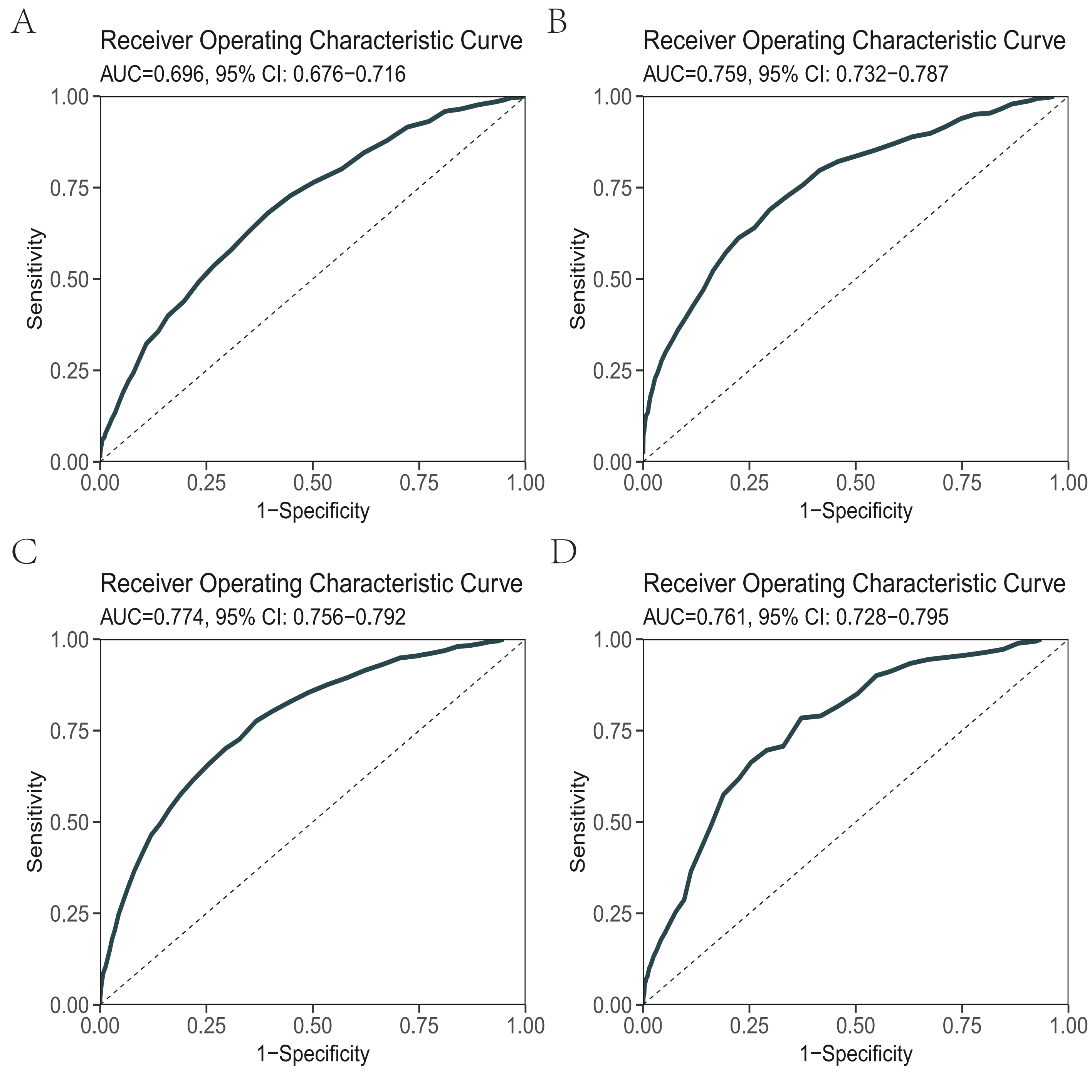

Supplement: Supplementary file 4 — Supporting information. [file CLC-48-e70101-s003.png]

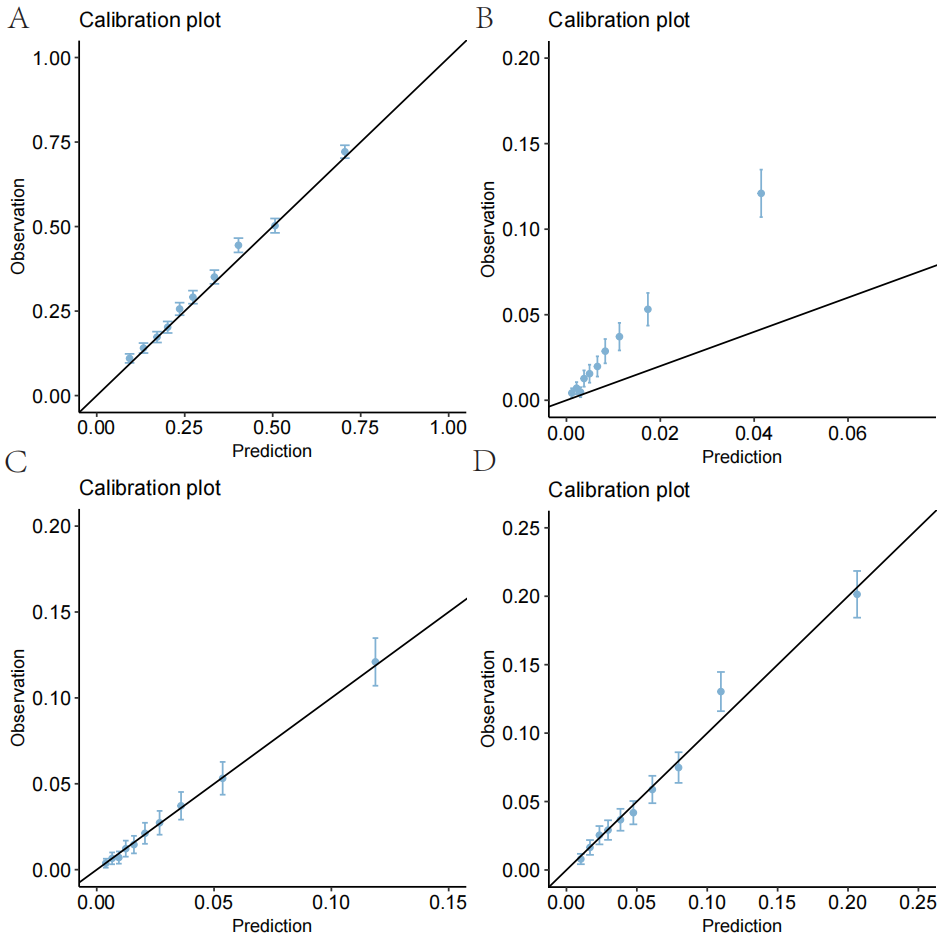

Supplement: Supplementary file 5 — Supporting information. [file CLC-48-e70101-s006.tif]

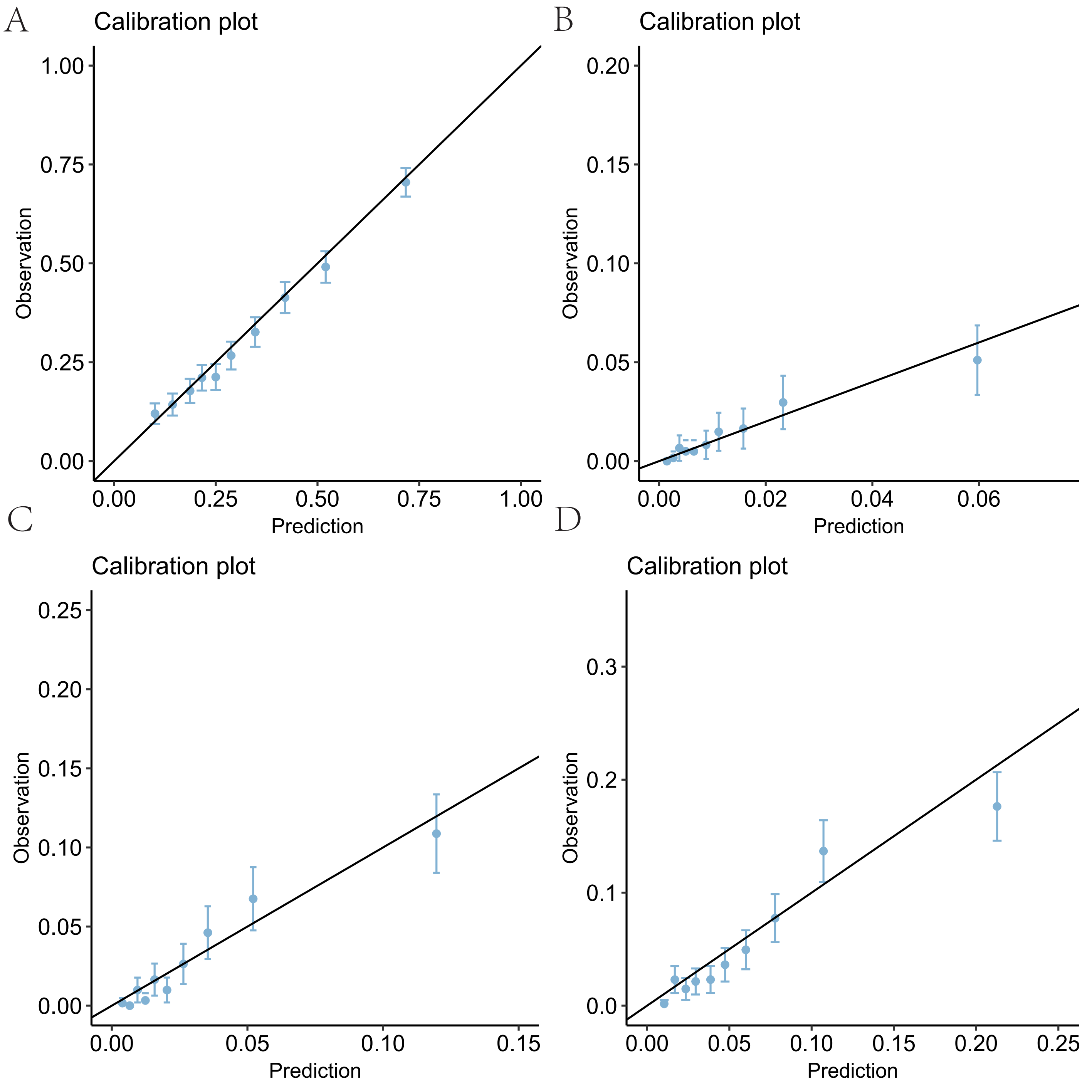

Supplement: Supplementary file 6 — Supporting information. [file CLC-48-e70101-s008.png]
