# Supplementary material for: The Scoring Model to Predict ICU Stay and Mortality After Emergency Admissions in Atrial Fibrillation: A Retrospective Study of 30 366 Patients
Source: Clin Cardiol. 2025 Feb 20;48(2):e70101. doi: 10.1002/clc.70101 (PMC11841604; doi:10.1002/clc.70101)
Supplement: Supplementary file 2 — Supporting information. [file CLC-48-e70101-s001.pdf]

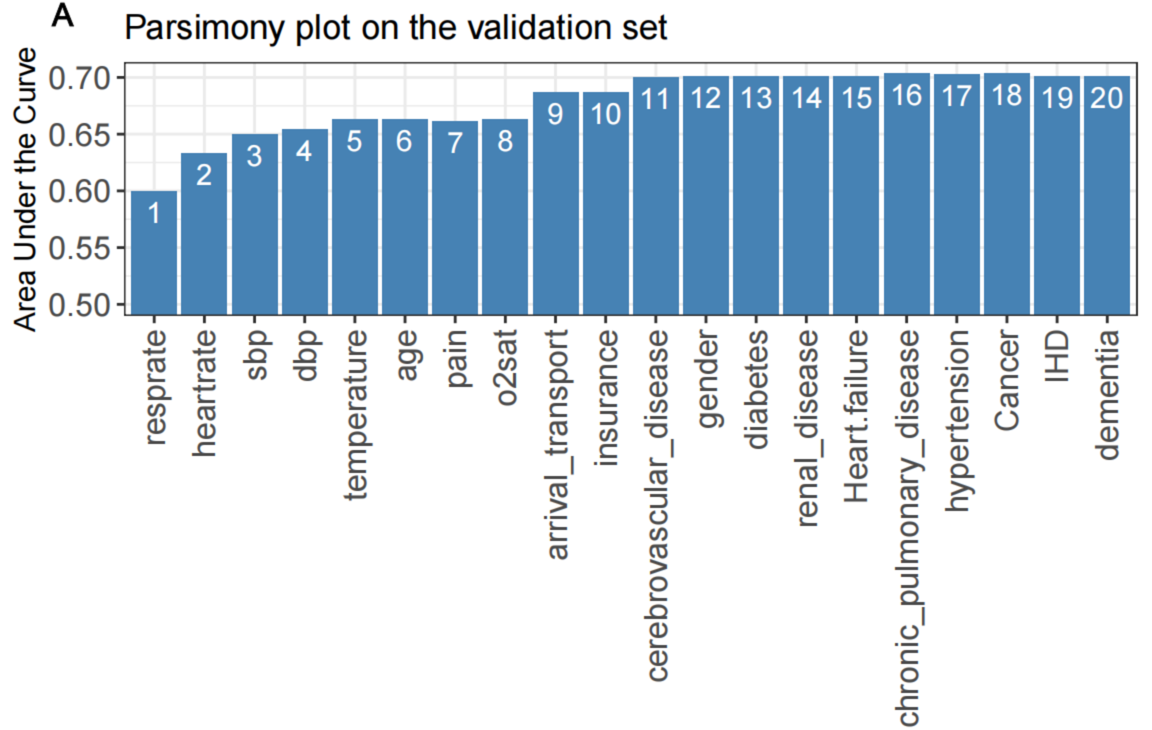
A
Parsimony plot on the validation set
Area Under the Curve
0.70
0.65
0.60
0.55
0.50
1
2
3
4
5
6
7
8
9
10
11
12
13
14
15
16
17
18
19
20
resprate
heartrate
sbp
dbp
temperature
age
pain
o2sat
arrival_transport
insurance
cerebrovascular_disease
gender
diabetes
renal_disease
Heart.failure
chronic_pulmonary_disease
hypertension
Cancer
IHD
dementia

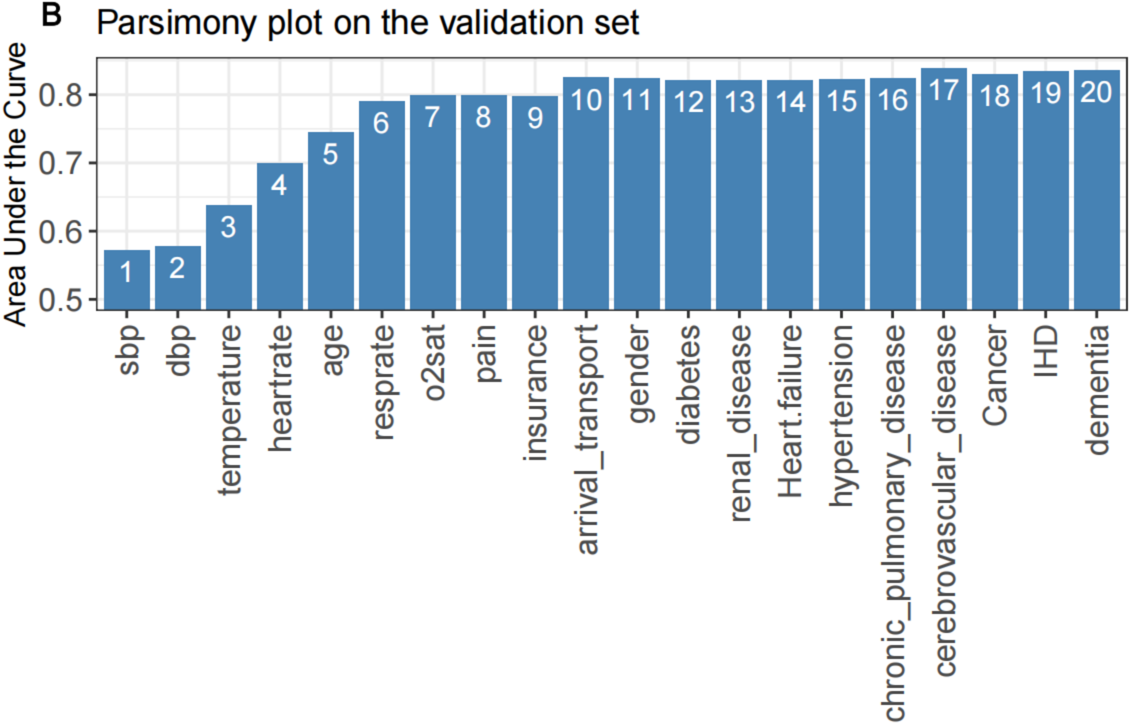
B
Parsimony plot on the validation set
Area Under the Curve
0.5
0.6
0.7
0.8
1
2
3
4
5
6
7
8
9
10
11
12
13
14
15
16
17
18
19
20
sbp
dbp
temperature
heartrate
age
resprate
o2sat
pain
insurance
arrival_transport
gender
diabetes
renal_disease
Heart.failure
hypertension
chronic_pulmonary_disease
cerebrovascular_disease
Cancer
IHD
dementia

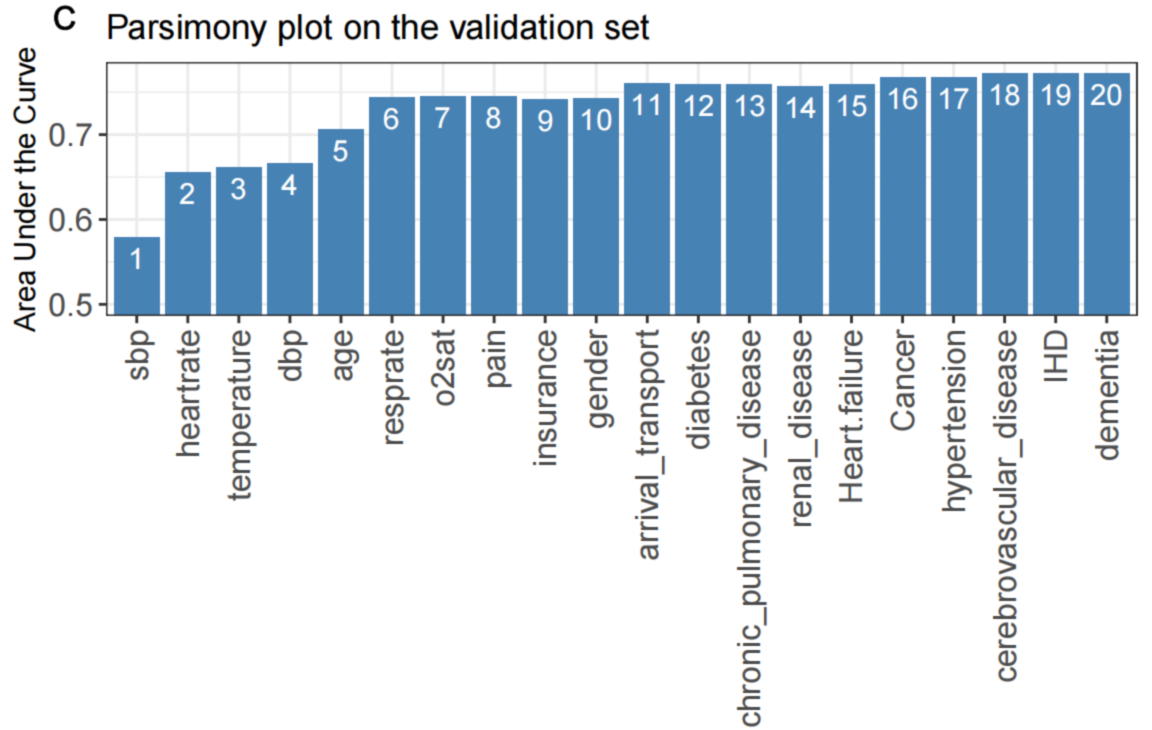
c
Parsimony plot on the validation set
Area Under the Curve
0.5
0.6
0.7
1
2
3
4
5
6
7
8
9
10
11
12
13
14
15
16
17
18
19
20
sbp
heartrate
temperature
dbp
age
resprate
o2sat
pain
insurance
gender
arrival_transport
diabetes
chronic_pulmonary_disease
renal_disease
Heart.failure
Cancer
hypertension
cerebrovascular_disease
IHD
dementia

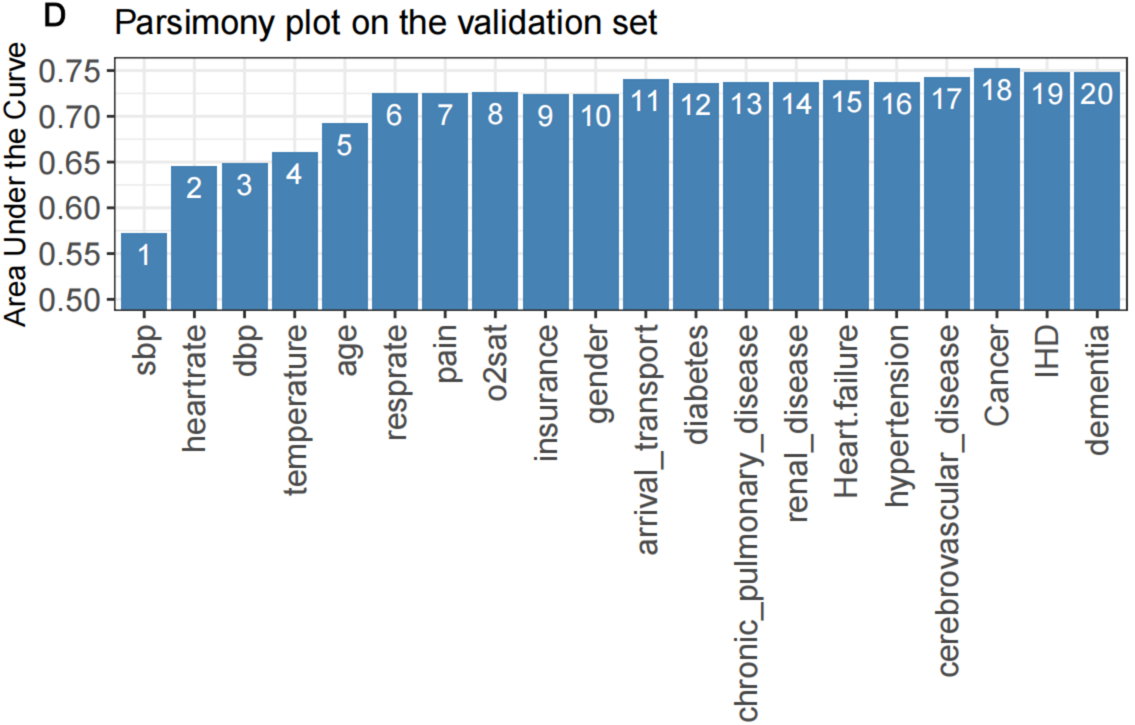
D
Parsimony plot on the validation set
Area Under the Curve
0.75
0.70
0.65
0.60
0.55
0.50
1
2
3
4
5
6
7
8
9
10
11
12
13
14
15
16
17
18
19
20
sbp
heartrate
dbp
temperature
age
resprate
pain
o2sat
insurance
gender
arrival_transport
diabetes
chronic_pulmonary_disease
renal_disease
Heart.failure
hypertension
cerebrovascular_disease
Cancer
IHD
dementia
