# Supplementary material for: The Scoring Model to Predict ICU Stay and Mortality After Emergency Admissions in Atrial Fibrillation: A Retrospective Study of 30 366 Patients
Source: Clin Cardiol. 2025 Feb 20;48(2):e70101. doi: 10.1002/clc.70101 (PMC11841604; doi:10.1002/clc.70101)
Supplement: Supplementary file 7 — Supporting information. [file CLC-48-e70101-s007.docx]

**SUPPLEMENTARY MATERIALS**


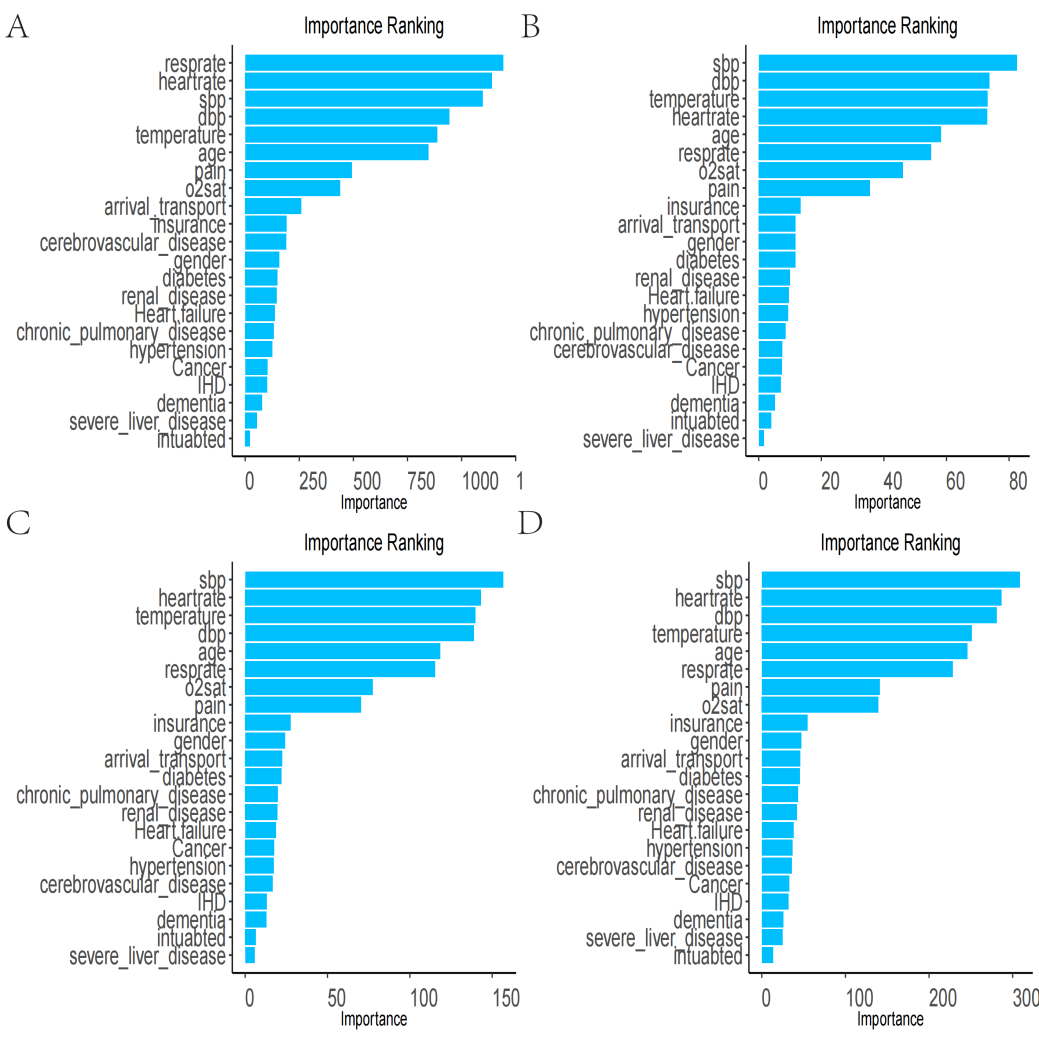


**Figure S1**. Feature importance ranking of all variables of the ICU stay (A), 3-day death (B), 7-day death (C), and 30-day death (D)

A

*
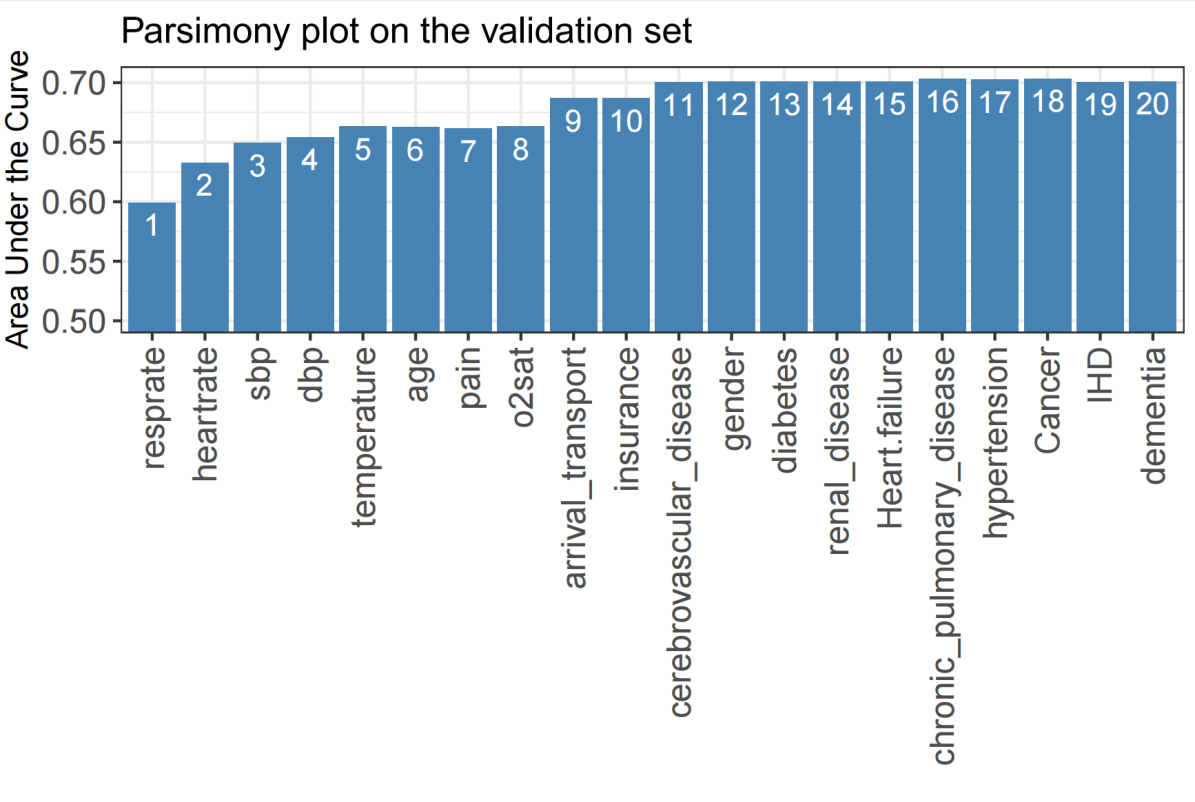
*

**B**

*
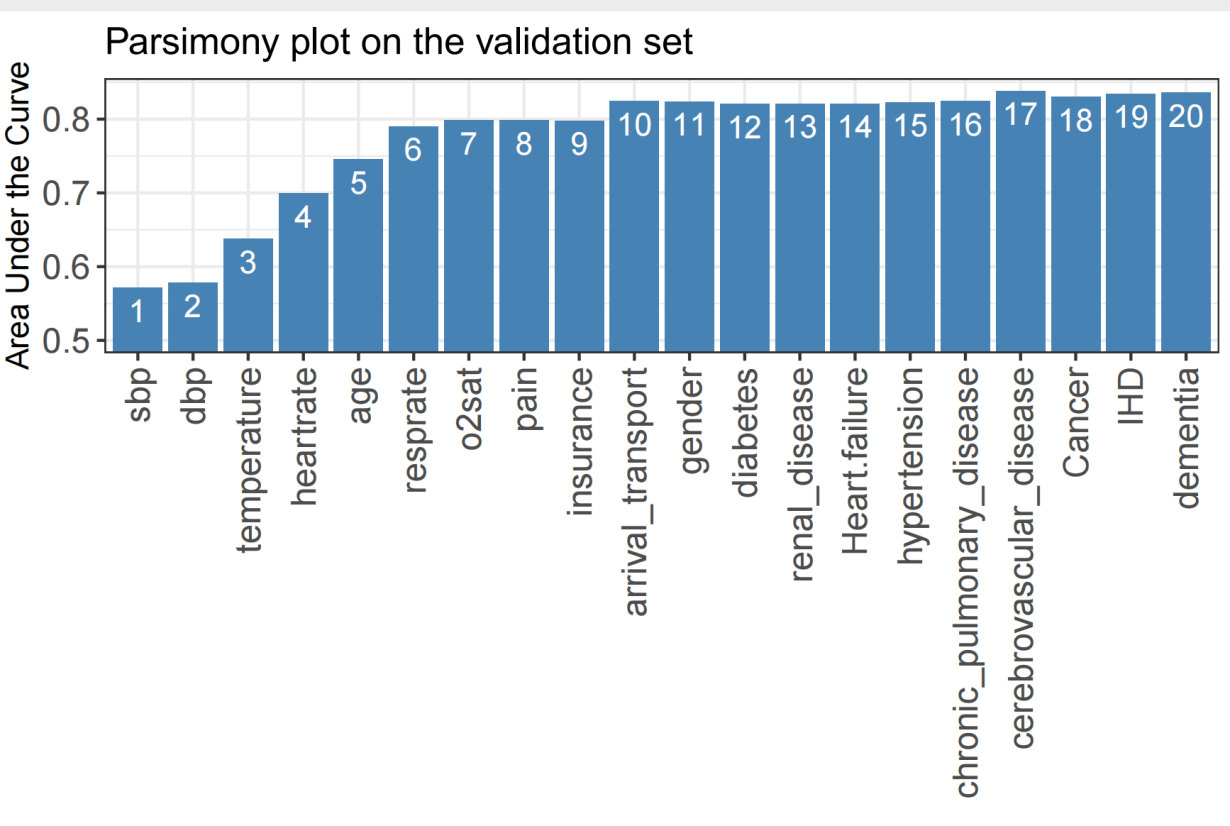
*

**C**

*
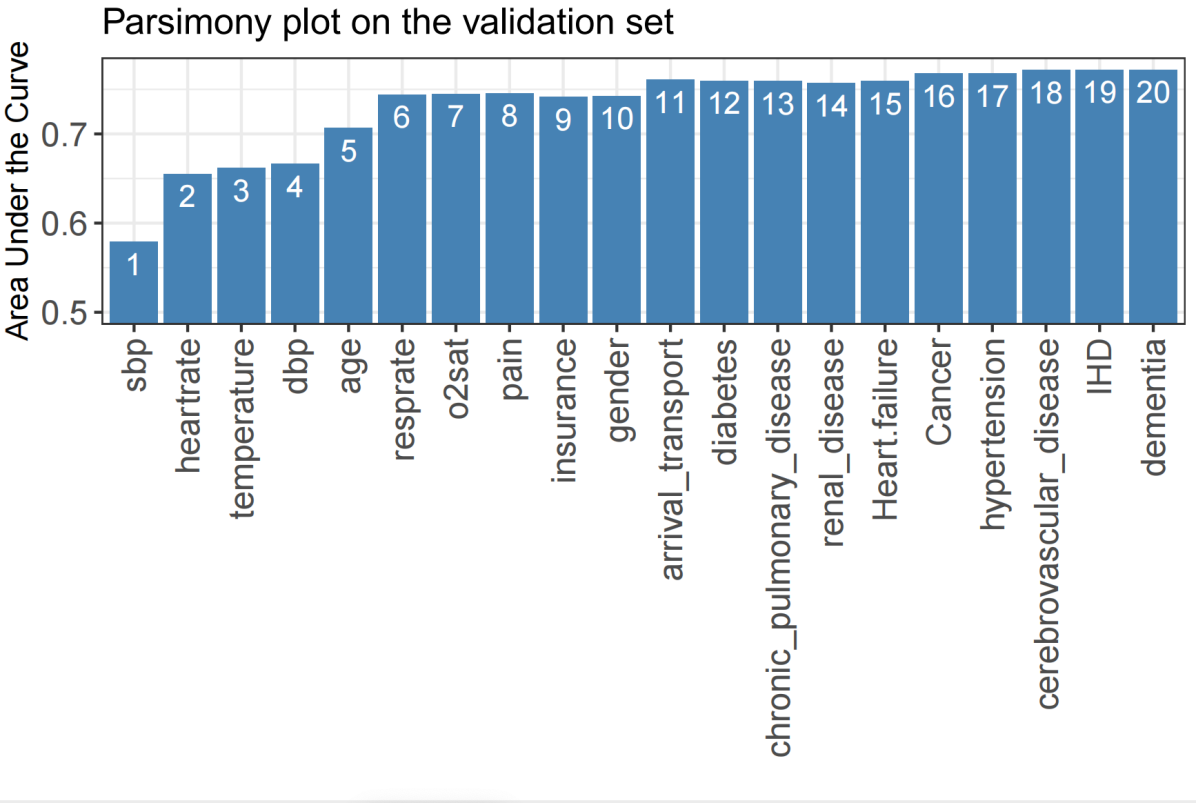
*

**D**

*
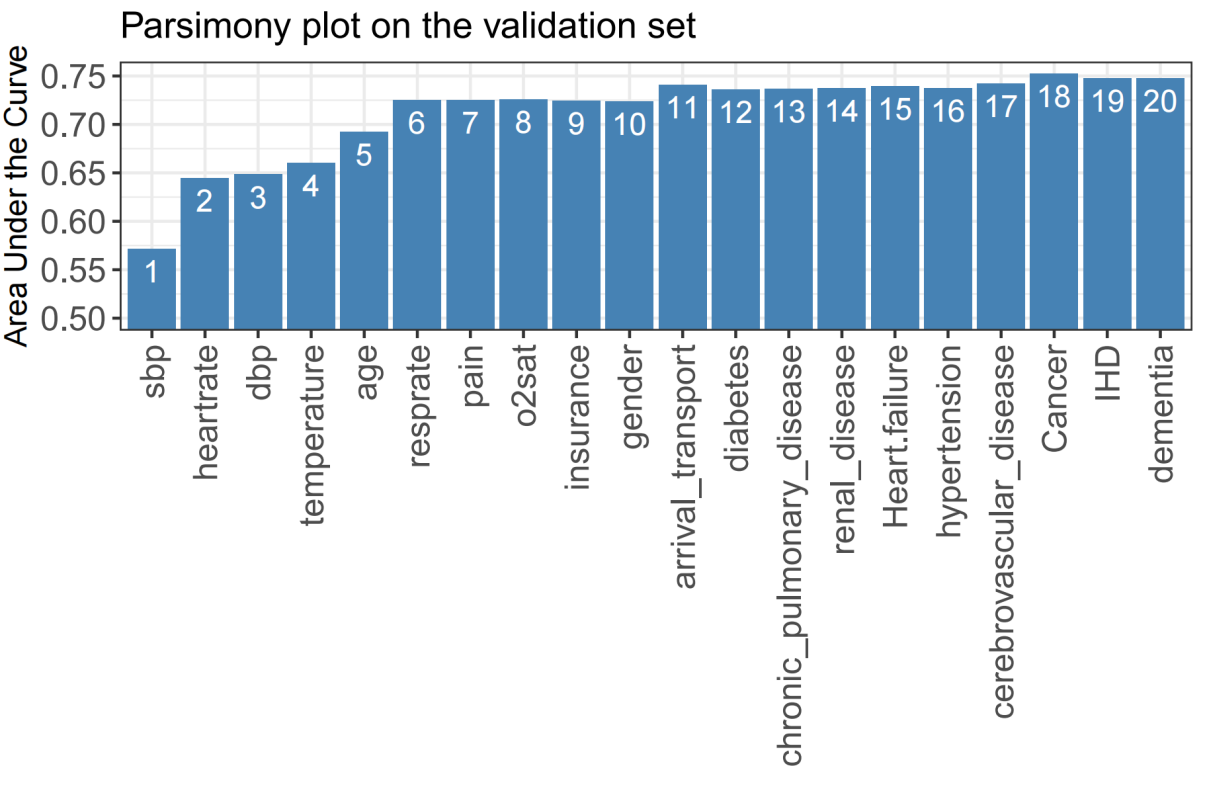
*

*Abbreviation: SBP, systolic blood pressure; DBP, diastolic blood pressure; ICU, intensive care unit; IHD, ischemic heart disease*

**Figure S2.** AUC of different variables included in the ICU stay (A), 3-day death (B), 7-day death (C), and 30-day death (D) in the validation set

**Table S1**. Varying risk probability of predicted risk based on the scoring models of ICU stay, 3-day death, 7-day death, and 30-day death in the testing cohort

| Outcome | Predicted Risk [>=] | Score cut-off [>=] | Percentage of patients (%) | Accuracy (95% CI) | Sensitivity (95% CI) | Specificity (95% CI) | PPV (95% CI) | NPV (95% CI) |
| --- | --- | --- | --- | --- | --- | --- | --- | --- |
| ICU stay | 5% | 2 | 100 | 30.7% (30.7-30.7%) | 100% (100-100%) | 0% (0-0%) | 30.7% (30.7-30.7%) | NA% (NA-NA%) |
|  | 10% | 8 | 95 | 34.5% (34-35%) | 98.5% (98-99.1%) | 6.1% (5.4-6.9%) | 31.7% (31.5-31.9%) | 90.6% (87-93.7%) |
|  | 20% | 16 | 66 | 54.3% (53.1-55.5%) | 82.6% (80.8-84.2%) | 41.8% (40.3-43.2%) | 38.5% (37.8-39.3%) | 84.4% (83-85.8%) |
|  | 30% | 21 | 39 | 68.4% (67.3-69.6%) | 62.5% (60.3-64.7%) | 71.1% (69.7-72.4%) | 48.9% (47.4-50.3%) | 81.1% (80.2-82%) |
|  | 50% | 29 | 14 | 73.8% (73-74.7%) | 30.2% (28.2-32.3%) | 93.1% (92.3-93.9%) | 66% (63.1-69.1%) | 75.1% (74.6-75.7%) |
|  | 75% | 39 | 3 | 71% (70.6-71.4%) | 6.9% (5.7-8%) | 99.4% (99.1-99.6%) | 82.8% (76.4-88.5%) | 70.7% (70.5-71%) |
| 3-day death | 0.10% | 9 | 99 | 2.7% (2.4-2.9%) | 100% (100-100%) | 1.3% (1-1.6%) | 1.4% (1.4-1.4%) | 100% (100-100%) |
|  | 0.50% | 23 | 67 | 33.8% (32.7-35.1%) | 91.7% (85.7-96.4%) | 33% (31.8-34.3%) | 1.9% (1.8-2%) | 99.6% (99.4-99.9%) |
|  | 1% | 30 | 38 | 63.1% (61.9-64.3%) | 81% (72.6-89.3%) | 62.8% (61.6-64.1%) | 3% (2.7-3.3%) | 99.6% (99.4-99.8%) |
|  | 1.50% | 33 | 27 | 73.6% (72.5-74.6%) | 67.9% (58.3-77.4%) | 73.7% (72.5-74.7%) | 3.5% (3-4%) | 99.4% (99.2-99.6%) |
|  | 3% | 39 | 10 | 89.4% (88.6-90.1%) | 36.9% (27.4-47.6%) | 90.1% (89.3-90.8%) | 4.9% (3.6-6.3%) | 99% (98.9-99.2%) |
|  | 5% | 44 | 3 | 95.9% (95.4-96.3%) | 26.2% (16.7-35.7%) | 96.8% (96.4-97.3%) | 10.4% (6.8-13.9%) | 98.9% (98.8-99.1%) |
| 7-day death | 1% | 19 | 67 | 35.4% (34.2-36.7%) | 95.5% (92-98.3%) | 33.6% (32.4-34.9%) | 4.1% (4-4.3%) | 99.6% (99.3-99.8%) |
|  | 2% | 25 | 41 | 60.7% (59.4-61.9%) | 86.4% (81.2-90.9%) | 59.9% (58.6-61.1%) | 6.1% (5.7-6.4%) | 99.3% (99.1-99.6%) |
|  | 5% | 33 | 15 | 85.5% (84.6-86.3%) | 52.8% (45.5-60.2%) | 86.5% (85.6-87.3%) | 10.4% (8.9-11.9%) | 98.4% (98.1-98.6%) |
|  | 10% | 39 | 5 | 93.9% (93.4-94.4%) | 23.3% (17-29.5%) | 96% (95.5-96.5%) | 14.9% (11.2-18.8%) | 97.7% (97.5-97.9%) |
|  | 12% | 41 | 3 | 95% (94.6-95.5%) | 17% (11.4-22.7%) | 97.4% (97-97.8%) | 16.2% (11.3-21.3%) | 97.5% (97.4-97.7%) |
|  | 15% | 43 | 2 | 96% (95.6-96.3%) | 13.6% (8.5-18.8%) | 98.4% (98.1-98.7%) | 20.5% (13.4-27.7%) | 97.4% (97.3-97.6%) |
| 30-day death | 1% | 10 | 93 | 12.6% (12-13.3%) | 100% (100-100%) | 7.5% (6.8-8.1%) | 6% (6-6.1%) | 100% (100-100%) |
|  | 5% | 26 | 36 | 67.3% (66.1-68.5%) | 75.3% (70.6-80%) | 66.8% (65.6-68%) | 11.9% (11.1-12.6%) | 97.9% (97.5-98.3%) |
|  | 10% | 33 | 14 | 85.8% (84.9-86.5%) | 43.8% (38.2-49.4%) | 88.3% (87.4-89%) | 18.1% (16.1-20.2%) | 96.4% (96-96.7%) |
|  | 15% | 37 | 6 | 90.6% (90-91.2%) | 22.6% (18.2-27.1%) | 94.7% (94.1-95.2%) | 20% (16.6-23.7%) | 95.4% (95.1-95.6%) |
|  | 20% | 41 | 3 | 93% (92.6-93.4%) | 12.6% (9.1-16.2%) | 97.8% (97.4-98.2%) | 25.3% (19.3-31.7%) | 95% (94.8-95.2%) |
|  | 50% | 54 | 0 | 94.4% (94.3-94.5%) | 2.4% (0.9-4.1%) | 99.9% (99.8-99.9%) | 50% (25-75%) | 94.5% (94.4-94.6%) |


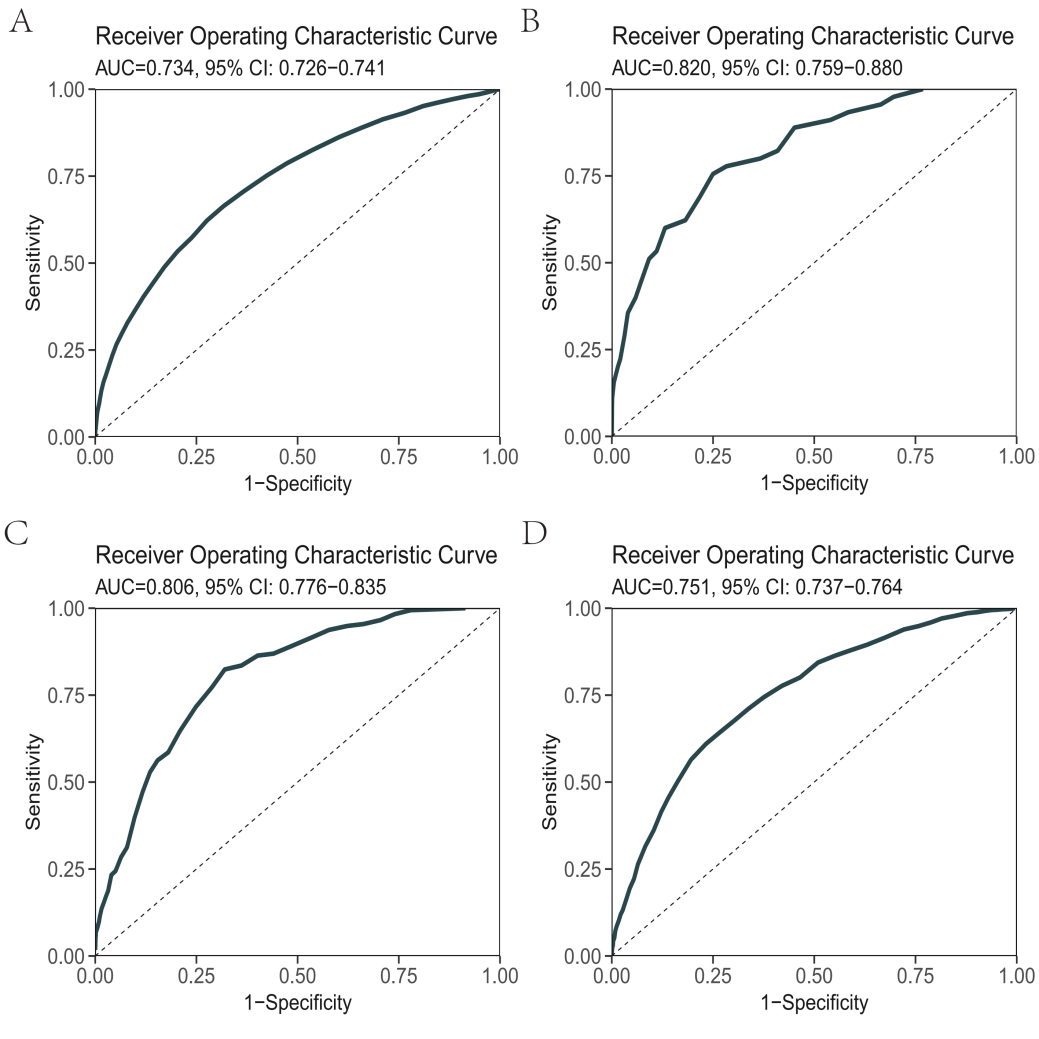


**Figure S3**. AUC values of ICU stay (A), 3-day death (B), 7-day death (C), and 30-day death (D) in the training set


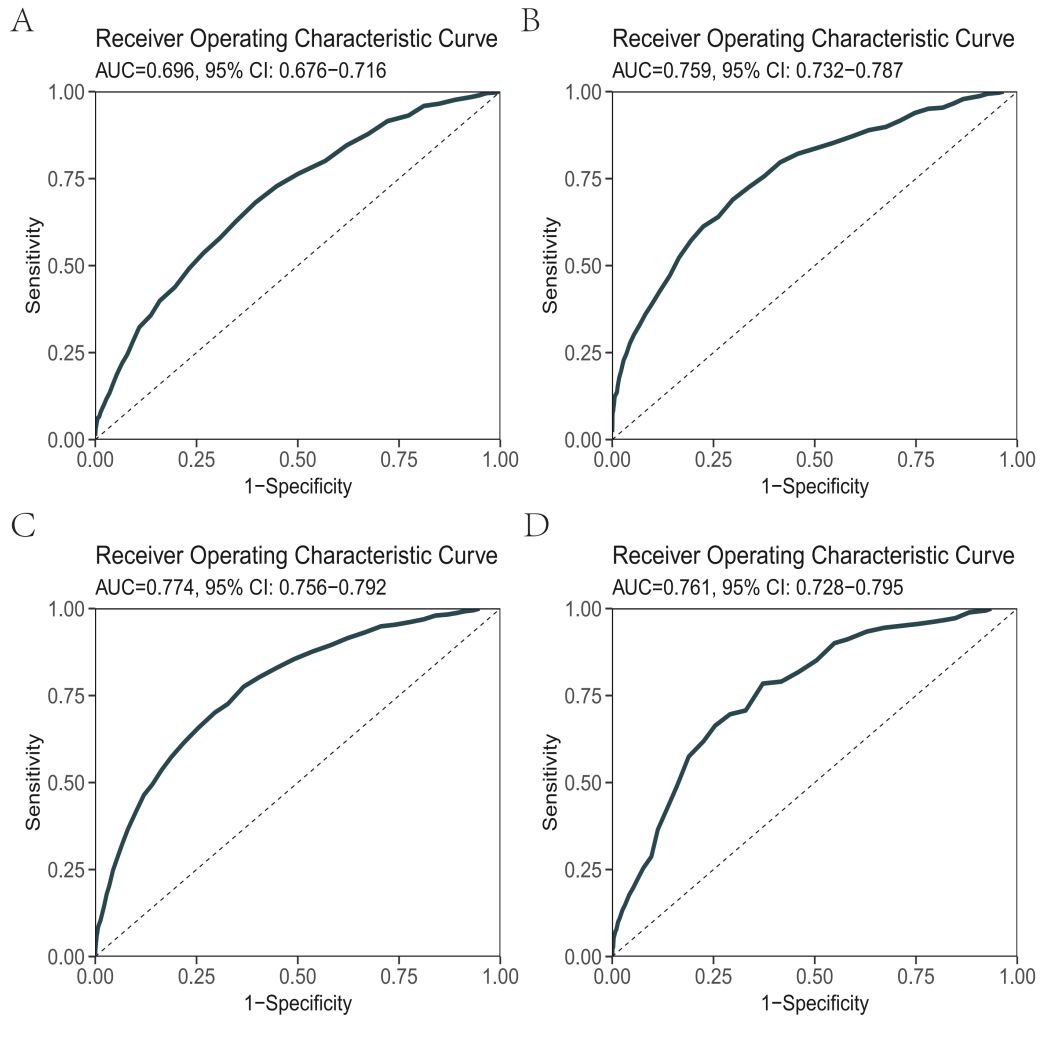
**Figure S4**. AUC values of the ICU stay (A), 3-day death (B), 7-day death (C), and 30-day death (D) in the validation set


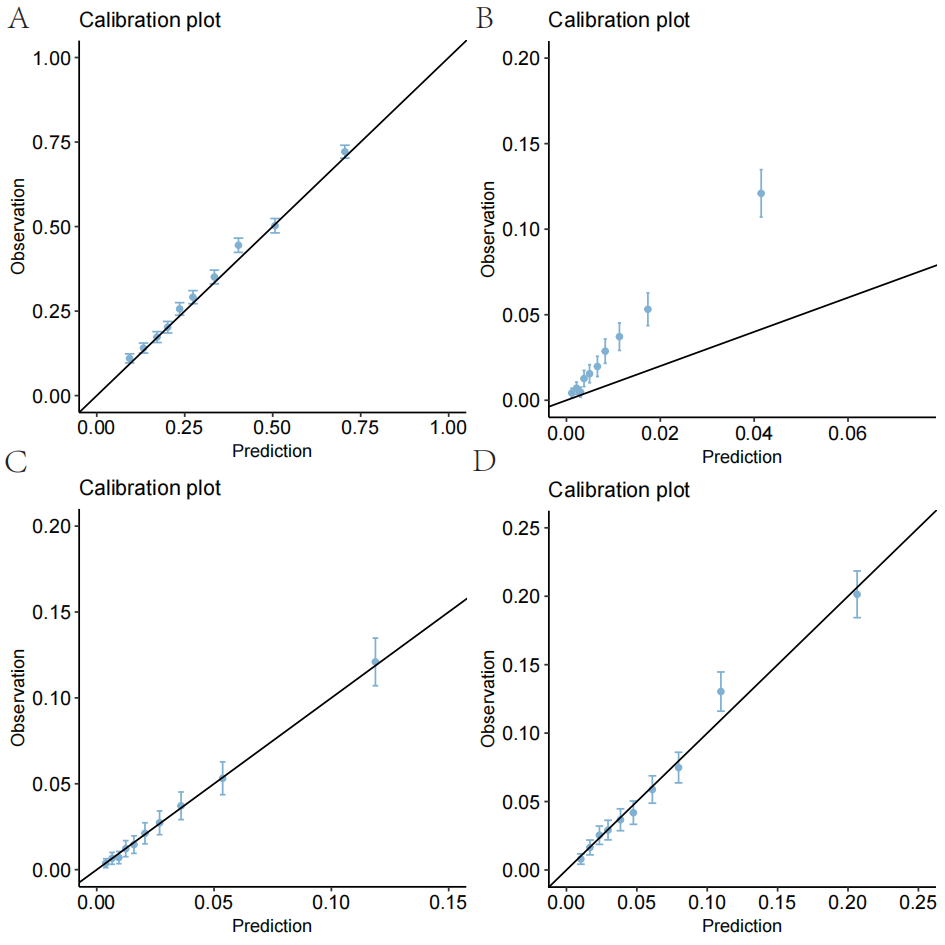


**Figure S5** Calibration curves of the ICU stay (A), 3-day death (B), 7-day death (C), and 30-day death (D) in the training set


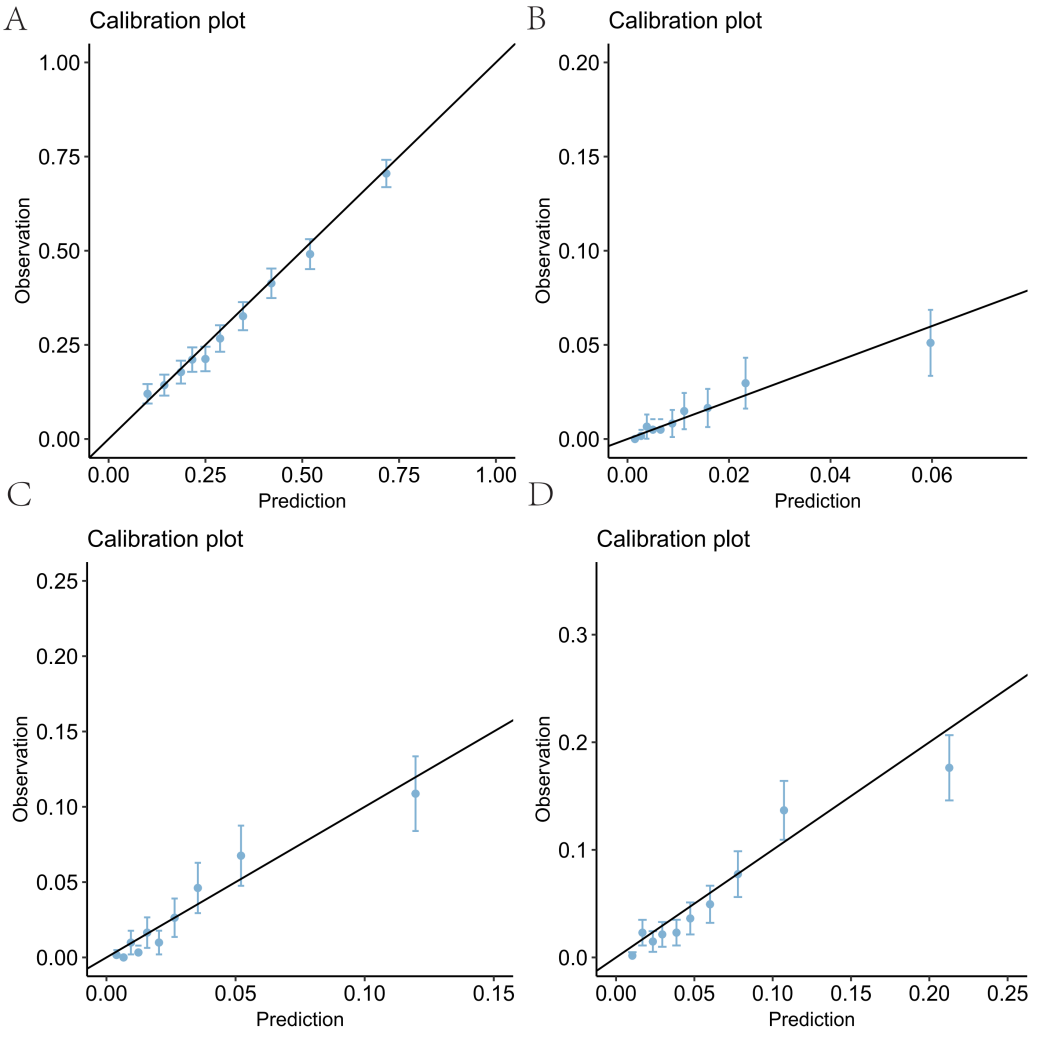


**Figure S6**. Calibration curves of the ICU stay (A), 3-day death (B), 7-day death (C), and 30-day death (D) in the testing set
