# Supplementary material for: The Scoring Model to Predict ICU Stay and Mortality After Emergency Admissions in Atrial Fibrillation: A Retrospective Study of 30 366 Patients
Source: Clin Cardiol. 2025 Feb 20;48(2):e70101. doi: 10.1002/clc.70101 (PMC11841604; doi:10.1002/clc.70101)
Supplement: Supplementary file 8 — Supporting information. [file CLC-48-e70101-s004.pdf]

Outcome	Predicted Risk [>=]	Score cut-off [>=]	Percentage of patients (%)	Accuracy (95% CI)	Sensitivity (95% CI)	Specificity (95% CI)	PPV (95% CI)	NPV (95% CI)
ICU stay	5%	2	100	30.7% (30.7-30.7%)	100% (100-100%)	0% (0-0%)	30.7% (30.7-30.7%)	NA% (NA-NA%)
	10%	8	95	34.5% (34-35%)	98.5% (98-99.1%)	6.1% (5.4-6.9%)	31.7% (31.5-31.9%)	90.6% (87-93.7%)
	20%	16	66	54.3% (53.1-55.5%)	82.6% (80.8-84.2%)	41.8% (40.3-43.2%)	38.5% (37.8-39.3%)	84.4% (83-85.8%)
	30%	21	39	68.4% (67.3-69.6%)	62.5% (60.3-64.7%)	71.1% (69.7-72.4%)	48.9% (47.4-50.3%)	81.1% (80.2-82%)
	50%	29	14	73.8% (73-74.7%)	30.2% (28.2-32.3%)	93.1% (92.3-93.9%)	66% (63.1-69.1%)	75.1% (74.6-75.7%)
	75%	39	3	71% (70.6-71.4%)	6.9% (5.7-8%)	99.4% (99.1-99.6%)	82.8% (76.4-88.5%)	70.7% (70.5-71%)
3-day death	0.10%	9	99	2.7% (2.4-2.9%)	100% (100-100%)	1.3% (1-1.6%)	1.4% (1.4-1.4%)	100% (100-100%)
	0.50%	23	67	33.8% (32.7-35.1%)	91.7% (85.7-96.4%)	33% (31.8-34.3%)	1.9% (1.8-2%)	99.6% (99.4-99.9%)
	1%	30	38	63.1% (61.9-64.3%	81% (72.6-89.3%	62.8% (61.6-64.1%)	3% (2.7-3.3%)	99.6% (99.4-99.8

				)	)			%)
	1.50%	33	27	73.6% (72.5-74.6%)	67.9% (58.3-77.4%)	73.7% (72.5-74.7%)	3.5% (3-4%)	99.4% (99.2-99.6%)
	3%	39	10	89.4% (88.6-90.1%)	36.9% (27.4-47.6%)	90.1% (89.3-90.8%)	4.9% (3.6-6.3%)	99% (98.9-99.2%)
	5%	44	3	95.9% (95.4-96.3%)	26.2% (16.7-35.7%)	96.8% (96.4-97.3%)	10.4% (6.8-13.9%)	98.9% (98.8-99.1%)
7-day death	1%	19	67	35.4% (34.2-36.7%)	95.5% (92-98.3%)	33.6% (32.4-34.9%)	4.1% (4-4.3%)	99.6% (99.3-99.8%)
	2%	25	41	60.7% (59.4-61.9%)	86.4% (81.2-90.9%)	59.9% (58.6-61.1%)	6.1% (5.7-6.4%)	99.3% (99.1-99.6%)
	5%	33	15	85.5% (84.6-86.3%)	52.8% (45.5-60.2%)	86.5% (85.6-87.3%)	10.4% (8.9-11.9%)	98.4% (98.1-98.6%)
	10%	39	5	93.9% (93.4-94.4%)	23.3% (17-29.5%)	96% (95.5-96.5%)	14.9% (11.2-18.8%)	97.7% (97.5-97.9%)
	12%	41	3	95% (94.6-95.5%)	17% (11.4-22.7%)	97.4% (97-97.8%)	16.2% (11.3-21.3%)	97.5% (97.4-97.7%)
	15%	43	2	96% (95.6-96.3%)	13.6% (8.5-18.8%)	98.4% (98.1-98.7%)	20.5% (13.4-27.7%)	97.4% (97.3-97.6%)

30-day death	1%	10	93	12.6% (12-13.3%)	100% (100-100%)	7.5% (6.8-8.1%)	6% (6-6.1%)	100% (100-100%)
	5%	26	36	67.3% (66.1-68.5%)	75.3% (70.6-80%)	66.8% (65.6-68%)	11.9% (11.1-12.6%)	97.9% (97.5-98.3%)
	10%	33	14	85.8% (84.9-86.5%)	43.8% (38.2-49.4%)	88.3% (87.4-89%)	18.1% (16.1-20.2%)	96.4% (96-96.7%)
	15%	37	6	90.6% (90-91.2%)	22.6% (18.2-27.1%)	94.7% (94.1-95.2%)	20% (16.6-23.7%)	95.4% (95.1-95.6%)
	20%	41	3	93% (92.6-93.4%)	12.6% (9.1-16.2%)	97.8% (97.4-98.2%)	25.3% (19.3-31.7%)	95% (94.8-95.2%)
	50%	54	0	94.4% (94.3-94.5%)	2.4% (0.9-4.1%)	99.9% (99.8-99.9%)	50% (25-75%)	94.5% (94.4-94.6%)
